# A genome-wide association study identifies risk loci for childhood acute lymphoblastic leukemia at 10q26.13 and 12q23.1

**DOI:** 10.1038/leu.2016.271

**Published:** 2016-11-11

**Authors:** J Vijayakrishnan, R Kumar, M Y R Henrion, A V Moorman, P S Rachakonda, I Hosen, M I da Silva Filho, A Holroyd, S E Dobbins, R Koehler, H Thomsen, J A Irving, J M Allan, T Lightfoot, E Roman, S E Kinsey, E Sheridan, P D Thompson, P Hoffmann, M M Nöthen, S Heilmann-Heimbach, K H Jöckel, M Greaves, C J Harrison, C R Bartram, M Schrappe, M Stanulla, K Hemminki, R S Houlston

**Affiliations:** 1 Division of Genetics and Epidemiology, The Institute of Cancer Research, Sutton, UK; 2 Division of Molecular Genetic Epidemiology, German Cancer Research Centre, Heidelberg, Germany; 3 Leukemia Research Group, Northern Institute for Cancer Research, Newcastle University, Newcastle upon Tyne, UK; 4 Department of Human Genetics, Institute of Human Genetics, University of Heidelberg, Heidelberg, Germany; 5 Department of Health Sciences, Epidemiology and Cancer Statistics Group, University of York, York, UK; 6 Department of Paediatric and Adolescent Haematology and Oncology, Leeds General Infirmary, Leeds, UK; 7 Medical Genetics Research Group, Leeds Institute of Biomedical & Clinical Sciences, University of Leeds, Leeds, UK; 8 Paediatric and Familial Cancer Research Group, Institute of Cancer Sciences, University of Manchester, St Mary's Hospital, Manchester, UK; 9 Institute of Human Genetics, University of Bonn, Bonn, Germany; 10 Department of Biomedicine, Human Genomics Research Group, University Hospital Basel, Basel, Switzerland; 11 Institute for Medical Informatics, Biometry and Epidemiology, University Hospital Essen, Essen, Germany; 12 Haemato-Oncology Research Unit, Division of Molecular Pathology, Institute of Cancer Research, Sutton, UK; 13 General Paediatrics, University Hospital Schleswig-Holstein, Kiel, Germany; 14 Department of Paediatric Haematology and Oncology, Hannover Medical School, Hannover, Germany; 15 Center for Primary Health Care Research, Lund University, Malmö, Sweden

## Abstract

Genome-wide association studies (GWASs) have shown that common genetic variation contributes to the heritable risk of childhood acute lymphoblastic leukemia (ALL). To identify new susceptibility loci for the largest subtype of ALL, B-cell precursor ALL (BCP-ALL), we conducted a meta-analysis of two GWASs with imputation using 1000 Genomes and UK10K Project data as reference (totaling 1658 cases and 7224 controls). After genotyping an additional 2525 cases and 3575 controls, we identify new susceptibility loci for BCP-ALL mapping to 10q26.13 (rs35837782, *LHPP*, *P*=1.38 × 10^−11^) and 12q23.1 (rs4762284, *ELK3*, *P*=8.41 × 10^−9^). We also provide confirmatory evidence for the existence of independent risk loci at 9p21.3, but show that the association marked by rs77728904 can be accounted for by linkage disequilibrium with the rare high-impact *CDKN2A* p.Ala148Thr variant rs3731249. Our data provide further insights into genetic susceptibility to ALL and its biology.

## Introduction

Acute lymphoblastic leukemia (ALL) is the major pediatric cancer in western countries, with B-cell precursor (BCP) ALL accounting for ~80% of ALL cases.^1^ Despite this, the etiology of ALL is poorly understood and although there is indirect evidence for an infective origin, no specific environmental risk factor has been identified.^2, 3^ Evidence for inherited predisposition to ALL is provided by the increased risk shown in siblings of cases independent of the concordance in monozygotic twins, which has an *in utero* etiology.^4^ Support for polygenic susceptibility to ALL has come from genome-wide association studies (GWASs).^5, 6, 7, 8, 9^ Although these studies have so far identified single-nucleotide polymorphisms (SNPs) at seven loci influencing BCP-ALL at 7p12.2 (*IKZF1*), 9p21.3 (*CDKN2A*, two risk loci), 10p12.2 (near *PIP4K2A*), 10p14 (*GATA3*), 10q21.2 (near *ARID5B*) and 14q11.2 (near *CEBPE*), statistical modeling using genome-wide complex trait analysis predicts that additional risk loci conferring modest effects should be identifiable by further GWAS.^10^

Recovery of untyped genotypes through imputation provides a mechanism of exploiting GWAS data sets to identify new risk alleles.^11^ In addition, it enables fine mapping and refinement of association signals, for example, in identification of the *CDKN2A* p.Ala148Thr variant rs3731249 (hg19 chr9:g.21970916 G>A) as contributing to the 9p21.3 association signal.^8^ Recently, the use of the 1000 Genomes Project and the UK10K projects as a combined reference panel has been shown to improve imputation accuracy compared with using the 1000 Genomes Project data alone.^12, 13^

Here, we report imputation using the 1000 Genomes and the UK10K Project data as reference and meta-analysis of two GWASs to identify new susceptibility alleles for BCP-ALL. After replication genotyping in three additional case–control series, we have identified new risk loci for BCP-ALL at 10q26.13 and 12q23.1. Our findings provide further insights into the genetic and biological basis of this hematological malignancy.

## Materials and Methods

### Ethics

Collection of samples and clinicopathological information from subjects was undertaken with informed consent in accordance with the Declaration of Helsinki and ethical board approval. Ethical committee approval was obtained for Medical Research Council UKALL97/99 trial by individual UK treatment centers and approval for UKALL2003 was obtained from the Scottish Multi-Centre Research Ethics Committee (REC:02/10/052).^14, 15^ Additional ethical approval was obtained under the auspices of the Childhood Leukaemia Cell Bank, the UK Childhood Cancer Study and University of Heidelberg.

### GWAS data

The UK-GWAS and German-GWAS data sets have been previously reported.^6, 7^ Briefly, the UK-GWAS was based on constitutional DNA (that is, remission samples) of 459 white BCP-ALL cases from the UK Childhood Cancer Study (UKCCS; http://www.ukccs.org/; 258 males; mean age at diagnosis 5.3 years); 342 cases from the UK Medical Research Council ALL 97/99 (1997–2002) trial (190 males; mean age of diagnosis 5.7 years) and 23 cases from the Northern Institute for Cancer Research (16 males). Genotyping was performed using Illumina Human 317 K arrays (Illumina, San Diego, CA, USA; Available at: http://www.illumina.com). For controls, we used publicly accessible data generated by the Wellcome Trust Case Control Consortium 2 (http://www.wtccc.org.uk/) from 2699 individuals in the 1958 British Birth Cohort (Hap1.2M-Duo Custom array data) and 2501 individuals from the UK Blood Service. The German-GWAS was comprised of 1155 cases (620 males; mean age at diagnosis 6.0 years) ascertained through the Berlin–Frankfurt–Münster (BFM) trials (1993–2004) genotyped using the Illumina Human OmniExpress-12v1.0 arrays. For controls, we used genotype data from 2132 healthy individuals from the Heinz Nixdorf Recall study; consisting of 704 individuals genotyped using Illumina HumanOmni1-Quad_v1 and 1428 individuals genotyped on Illumina Human OmniExpress-12v1.0 platform. In total, we obtained 1658 BCP-ALL cases and 7224 matched controls from the two GWASs series combined.

### Quality control of GWAS samples

The quality-control steps of UK- and German-GWAS study samples have been described in the previous studies.^6, 7^ After the quality-control steps, we obtained 824 cases and 5200 controls for the UK-GWAS data set, and 834 cases and 2024 controls from the German data sets that were then used for further genotyping and imputation analysis.

### Replication series and genotyping

The UK replication series comprised 1150 patients (504 males; mean age at diagnosis 6.2 years) ascertained through the UK ALL-2003 (2003–2011) and ALL 97/99 trials.^14, 15^ Immunophenotyping of diagnostic samples was undertaken using standard methods. The 2100 controls (702 males) were ethnically-matched healthy individuals with no personal history of cancer recruited to the National Study of Colorectal Cancer Genetics^16^ and the Genetic Lung Cancer Predisposition Study.^17^ Genotyping of cases and controls was performed using competitive allele-specific PCR KASPAR chemistry (LCG Biosciences Ltd, Hertfordshire, UK). The German replication series consisted of 1501 patients ascertained (794 males; mean age at diagnosis, 6.2 years ascertained through the BFM trials (1993–2004)).^18^ The controls comprised of 1516 (762 males; mean age, 58.2 years), ethnically matched healthy individuals of German origin recruited at the Institute of Transfusion Medicine in Manheim, Germany, 2004. Samples having SNP call rates of <90% were excluded from the analysis. To ensure quality of genotyping in all assays, at least 2 negative controls and 1 to 2% duplicates (concordance >99.9%) were genotyped. All primers and probes used are detailed in Supplementary Table S11. Combining both replication series, we had access to 2651 B-cell ALL cases and 3616 matched controls for the current study.

### Sanger sequencing

To confirm the fidelity of imputation, a random subset of samples were sequenced using BigDye Terminator v3.1 Cycle Sequencing Kit (Applied Biosystems, Waltham, MA, USA) in conjunction with ABI 3700xl semi-automated sequencers (Applied Biosystems). Primer sequences are detailed in Supplementary Table S11.

### Statistical and bioinformatics analyses

Main data analysis were undertaken using R version 2.15.2 (R Core Team, 2013; http://www.R-project.org/), PLINK v1.9(ref. 19) and SNPTEST v2.4.1 software.^20^ The two GWAS data sets were imputed for over 10 million variants using IMPUTE2 v2.3.0 software^21, 22^ and data from the 1000 Genomes Project (phase 1 integrated variant set, v3.20101123, http://www.1000genomes.org, 9 December 2013) and UK10K (ALSPAC, EGAS00001000090/EGAD00001000195; and TwinsUK, EGAS00001000108/EGAD00001000194, studies only; http://www.uk10k.org/) as reference. Data sets were first phased using SHAPEIT v2.12 prior to imputation to accurately estimate haplotypes.^23^ The adequacy of case–control matching and possibility of differential genotyping between cases and controls were evaluated using quantile–quantile plots of test statistics to compute *λ*_100_. Test of association between imputed SNPs and childhood ALL was performed using a missing data likelihood score test under a frequentist additive model in software SNPTEST. Eigenvectors for the German data set were inferred using *smartpca* component within EIGENSOFT v2.4(refs. 24, 25) and Eigenstrat adjustment was carried out by including the first two eigenvectors as covariates in SNPTEST during association analysis. Post imputation and SNPTEST, only markers with info scores >0.4, imputed call rates/SNP >0.9, minor allele frequencies (MAFs) >0.005, and a posterior imputation quality threshold of 0.5 or higher were included in further analysis. SNPs that deviated from Hardy–Weinberg equilibrium at *P*-values <10^−5^ were also excluded from further analysis. Meta-analysis of post quality control GWAS data sets was conducted in META 1.3.1,^20, 21, 26^ under a fixed-effects model using the inverse variance approach. We calculated Cochran's *Q* statistic to test for heterogeneity and the *I*^2^ statistic to quantify the proportion of the total variation attributable to heterogeneity.^27^ The presence of secondary association signals owing to allelic heterogeneity in risk loci were carried out using a conditional analysis in SNPTEST by adjusting for the sentinel SNP using the ‘–condition-on' option. Logistic regression association analysis and meta-analysis of the replication data sets under fixed effects were carried out using the STATA v.10 software (Stata Corporation, College Station, TX, USA).

Linkage disequilibrium (LD) metrics were calculated using vcftools v0.1.12b26 (http://vcftools.sourceforge.net) using UK10K data. HapMap recombination rate (cM/Mb) were defined by Oxford recombination hotspots.^28, 29^

### Chromatin state dynamics and functional annotation

To explore the epigenetic profile of association signals, we used 15-state chromatin segmentation data learned by computationally integrating chIP-seq data for GM12878 lymphoblastoid cells inferred from ENCODE Histone Modification data (H4K20me1, H3K9ac, H3K4me3, H3K4me2, H3K4me1, H3K36me3, H3K27me3, H3K27ac and CTCF) and binarized using a multivariate Hidden Markov Model (http://genome.ucsc.edu/ENCODE/).^30^ Risk SNPs and their proxies (that is, *r*^2^>0.8 in the 1000 Genomes EUR reference panel) were annotated for putative functional effect using HaploReg v3,^31^ RegulomeDB^32^ and SeattleSeq^33^ Annotation. These servers make use of data from ENCODE,^30^ genomic evolutionary rate profiling^34^ conservation metrics, combined annotation-dependent depletion scores^35^ and PolyPhen scores.^36^ Similarly, we searched for overlap with ‘super-enhancer' regions as defined by Hnisz *et al.*^37^ restricting analysis to GM12878 cells.

### Expression quantitative trait locus analysis

Expression quantitative trait locus (eQTL) analysis was performed for all genes in 1 Mb regions spanning rs4762284 and rs35837782 by querying messenger RNA expression data from MuTHER^38^ and Blood eQTL browser.^39^

### Chromosome karyotyping and 9p21.3 deletion status

Conventional cytogenetic studies on diagnostic ALL tumor cells were conducted using standard karyotyping methodologies, and standard criteria for the definition of a clone were applied. Genomic copy number at 9p21.3 was assayed using FISH and MLPA as previously described.^40, 41^

### Relationship between SNP genotype and survivorship

To investigate if genotype is associated with clinical phenotype or outcome, we analyzed data on patients recruited to AIEOP-BFM 2000.^18^ Briefly, patients received standard chemotherapy (that is, prednisone, vincristine, daunorubicin, l-asparaginase, cyclophosphamide, ifosfamide, cytarabine, 6-mercaptopurine, 6-thioguanine and methotrexate) with a subset of high-risk patients treated with cranial irradiation and/or stem cell transplantation. Event-free survival was defined as the time from diagnosis to the date of last follow-up in complete remission or to the first event. Events were resistance to therapy (nonresponse), relapse, secondary neoplasm or death from any cause. Failure to achieve remission owing to early death or nonresponse was considered as an event at time zero and patients lost to follow-up were censored at the time of their withdrawal. Patients were stratified into three categories: standard, intermediate and high risk. Although minimal residual disease analysis was the main stratification criterion, high risk was also defined by prednisone poor response or ⩾5% leukemic blasts in bone marrow on day 33, or *t*(9;22)/*t*(4;11) positivity or their molecular equivalents (BCR-ABL/MLL-AF4-fusion) independent of minimal residual disease status. Standard patients were minimal residual disease-negative on treatment day 33 (TP1) and 78 (TP2), and had no high-risk criteria. High-risk patients were defined as having residual disease (⩾10^−^^3^ cells) at TP2. Intermediate patients had positive-minimal residual disease detection at either TP1 or TP2, but had a cell count of <10^−^^3^ at TP2. The Kaplan–Meier method was used to estimate survival rates, differences were compared with the two-sided log-rank test.^42, 43^ Cumulative incidence functions for competing events were constructed by the method of Kalbfleisch and Prentice,^44^ and were compared employing the Gray's test.^45^ Computations were performed using SASv9.1 (SAS, Cary, NC, USA).

### Heritability analysis

We used genome-wide complex trait analysis to estimate the polygenic variance (that is, heritability) ascribable to all GWAS SNPs.^46^ SNPs were excluded based on the MAF (<0.01), missing genotype rate (0.05) and deviation from Hardy–Weinberg equilibrium (*P*<0.05). Individuals were excluded for exhibiting an excess of missing genotype (>0.02) and where two individuals were closely related (genetic relatedness score >0.05). A genetic relationship matrix of pairs of samples was used as input for the restricted maximum likelihood analysis to estimate the heritability explained by the selected set of SNPs. Regions of high LD in the genome were excluded from the analysis. Imposing a prevalence of 0.0005(ref. 2) for childhood ALL, we estimated the heritability explained by risk SNPs identified by GWAS as located within autosomal regions associated with ALL. For each risk SNP, the heritability was estimated for all chromosomes simultaneously using the risk SNP genotype as a covariate. In chromosomes bearing multiple independent risk loci, all the risk SNPs in that chromosome were used as covariates to get the combined contribution of risk SNPs toward heritability. The heritability associated with the risk SNPs was taken to be the difference between the heritability of the chromosome on which it is found as calculated with and without covariate adjustment for the SNP.

### Calculation of polygenic risk scores

In addition to the two new risk loci described here, seven previously reported risk loci were included in the calculation of the polygenic risk scores for childhood ALL (rs10828317, 10p12.2; rs3824662, 10p14; rs7089424, 10q21.2; rs2239633, 14q11.2; rs4132601, 7p12.2; rs3731249, 9p21.3; rs3731217, 9p21.3; rs35837782, 10q26.13; rs4762284, 12q23.1). The eight variants are thought to act independently as previous studies have shown no interaction between risk loci.^5, 6, 7^ Polygenic risk scores were constructed using methods established by Pharoah *et al.*^47^ based on the log-normal distribution LN(*μ*,*σ*^2^) of mean *μ* and variance *σ*^2^ (that is, relative risk is normally distributed on a logarithmic scale). Standardized incidence ratios for familial risk in singleton siblings and twins for childhood ALL were assumed to be 3.2.^4^ Familial risk was calculated by dividing polygenic variation over the square root of familial risk.

## Results

### Association analysis

To identify new susceptibility loci for BCP-ALL, we conducted a pooled meta-analysis of two GWASs in populations of European ancestry, the UK-GWAS and the German-GWAS (see Materials and Methods section). After filtering, the studies provided genotype data on 1658 cases and 7224 controls. To achieve consistent and dense genome-wide coverage, we imputed unobserved genotypes at >10 million SNPs using a combined reference panel comprising 1092 individuals from the 1000 Genomes Project and 3781 individuals from the UK10K project (Supplementary Figure S2). Quantile–quantile plots of SNPs (MAF >0.5%) post imputation did not show evidence of substantive overdispersion introduced by imputation (genomic inflation *λ*_100_ for UK- and German-GWAS was 1.016 and 1.009, respectively; Supplementary Figure S1).

Pooling data from both GWASs, we derived joint odds ratios and 95% confidence intervals under a fixed-effects model for each SNP with MAF >0.5% and associated per allele *P*-values. From this analysis, we identified the top-ranked SNPs in 20 distinct regions and not previously implicated in the risk of developing BCP-ALL (Supplementary Table S1). After confirming the fidelity of imputation by Sanger sequencing (Supplementary Table S2), we successfully designed and optimized allele-specific PCR (KASPAR) assays for 14 SNPs. We sought validation of associations by genotyping additional UK and German case–control series totaling 2525 cases and 3575 controls (Supplementary Table S3).

In the combined analysis of data from these replication series, rs35837782 (10q26.13, hg19 chr10:g.126293309) and rs4762284 (12q23.1, hg19 chr12:g.96612762) showed significant support for an association with BCP-ALL, with *P*-values and odds ratios of 3.66 × 10^−6^, 1.20 and 3.88 × 10^−4^, 1.16, respectively (Table 1; Supplementary Table S4; Supplementary Figure S3). In a meta-analysis of the discovery GWAS and replication series, these associations attained genome-wide significance (rs35837782, *P*=1.38 × 10^−11^ and rs4762284, *P*=8.41 × 10^−9^; Table 1; Supplementary Table S4; Supplementary Figure S3).

### Conditional association analyses

To explore the possibility of multiple risk loci at 10q26.13 and 12q23.1 and previously identified GWAS risk loci, we performed conditional analyses. At 10q26.13 and 12q23.1, we found no evidence for signals independent of SNPs rs35837782 and rs4762284. Similarly at 7p12.2, 10p12.2, 10p14, 10q21.2 and 14q11.2, we found no support for the existence of multiple risk loci.

We and others have recently sought to decipher the GWAS signal at the 9p21.3 locus.^8, 48, 49, 50^ Our conditional analysis supports the assertion of an additional locus in this region, independent of the original GWAS SNP rs3731217, which is best captured by the rare coding SNP rs3731249 (MAF=0.03, *r*^2^=0.005, *D*′=1.00 with rs3731217; Supplementary Table S5). rs3731249, encoding *CDKN2A* p.Ala148Thr, has been shown to reduce tumor suppressor function of p16INK4A, increase susceptibility to leukemic transformation of hematopoietic progenitor cells and to be preferentially retained in ALL cells.^49^ The more common variant rs662463 correlated with rs77728904 has concurrently been suggested as a plausible causative variant underlying this new association signal^48^ (MAF=0.07, *r*^2^=0.16, *D*′=1.00 with rs3731249). Despite some evidence that rs77728904 variant is a *cis*-eQTL for *CDKN2B*,^48^ this association signal is entirely captured by rs3731249 (*P*-values before and after conditioning: 6.26 × 10^−7^and 0.10, respectively; Supplementary Table S5). Here, our analysis has been constrained to the identification of variants that can be imputed with high fidelity, hence it does not exclude the possibility of rarer variants with higher impact, especially indels potentially impacting on ALL risk. This exemplifies the difficultly in elucidating the genetic basis of such functionally rich genomic regions. Once correcting for these two signals, no additional statistically significant association was detected in this region.

### Relationship between the new ALL-risk SNPs and tumor profile

Given the biological heterogeneity of BCP-ALL, we analyzed the association between rs35837782 and rs4762284 genotypes, and the major subtypes of BCP-ALL, hyperdiploidy (that is, >50 chromosomes), *ETV6-RUNX1* and others (Supplementary Table S6; Supplementary Figure S3). Analysis of these data provided no consistent evidence that the risk of rs35837782 and rs4762284 was confined to hyperdiploid, *ETV6-RUNX1* or non-hyperdiploid/non-*ETV6-RUNX1* subtypes of B-ALL. Similarly, we found no evidence for a relationship between rs35837782 and rs4762284 genotypes, and other chromosomally defined forms of BCP-ALL defined by *t*(9;22)(q34;q11), *t*(1;19)(q23;p13) and *t*(4;11)(q21;q23) karyotype, or *CDKN2A* deletion status after adjustment for multiple testing (Supplementary Table S6). Finally, we found no evidence that rs35837782 and rs4762284 genotypes were associated with age at diagnosis or sex, or influenced patient outcome as defined by event-free survival by analyzing data on 810 patients from the AIEOP-BFM 2002 trial (Supplementary Figure S4, Supplementary Table S7 and S8).

### Impact on the heritable risk

By fitting all SNPs from GWAS simultaneously, the estimated heritability of ALL attributable to all common variation is 12.1% (±3.8%). This estimate represents the additive variance, and therefore, does not include the potential impact of gene–gene interactions or dominance effects, or gene–environment interactions impacting on ALL risk. Moreover, given the evidence, albeit indirect, of a role for infectious exposure in relation to ALL risk, it is possible that substantive gene–environment effects operate. Although the currently identified risk SNPs (newly discovered and previously identified) only account for 19% of the additive heritable risk, the odds ratio effect sizes of the ALL-risk SNPs are among the highest reported in GWAS of any cancer type, and in combination they impact significantly on disease risk with those in the top 1% of genetic risk having a 6.2-fold relative risk of developing ALL (Supplementary Figure S5). The power of our GWAS to identify common alleles conferring relative risks of 1.5 or greater (such as the 7p12.2 variant) is high (~80%). Hence, there are unlikely to be many additional SNPs with similar effects for alleles with frequencies >0.3 in populations of European ancestry. In contrast, our analysis had limited power to detect alleles with smaller effects and/or MAF <0.1.

### Biological inference

**Figure 1:**
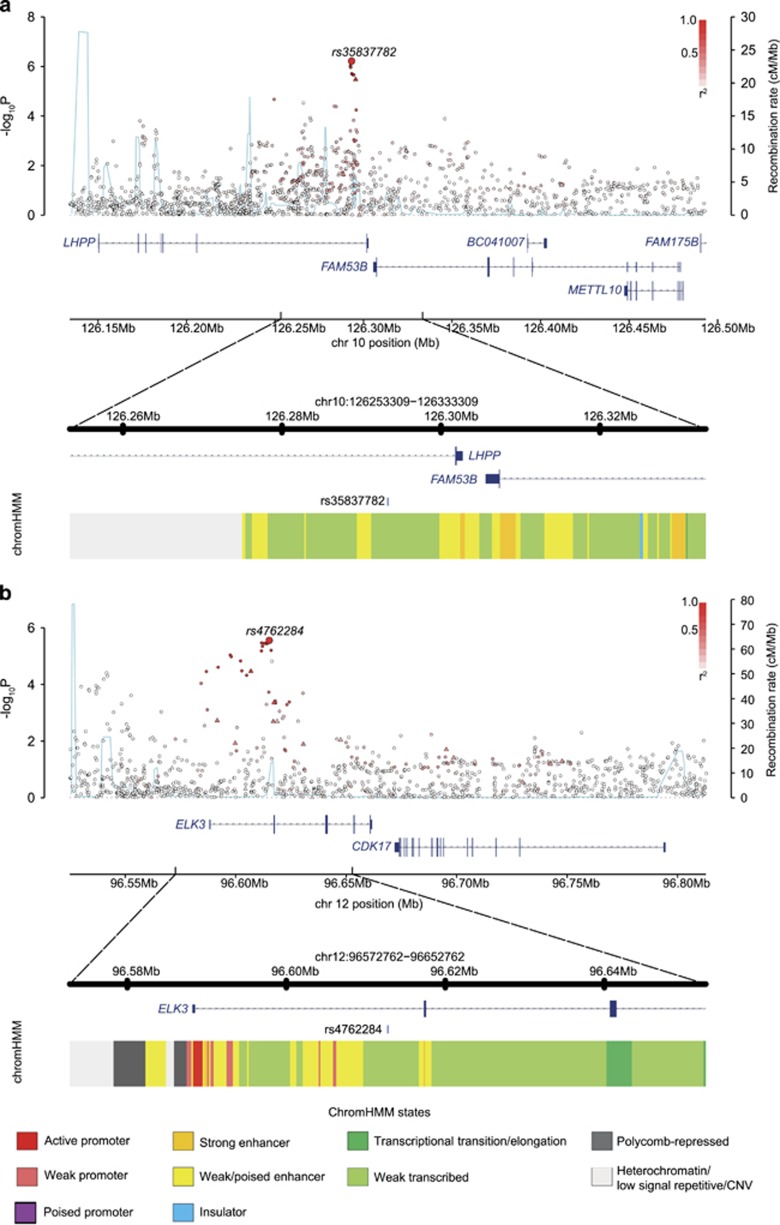
Regional plots of association results and recombination rates for the newly identified risk loci for BCP-ALL (**a** and **b**). Results for 10q26.13 (rs35837782, **a**) and 12q23.1 (rs4762284, **b**). Plots (using visPig)^56^ show association results of both genotyped (triangles) and imputed (circles) SNPs in the GWAS samples and recombination rates. −log10 *P*-values (*y* axes) of the SNPs are shown according to their chromosomal positions (*x* axes). The sentinel SNP in each combined analysis is shown as a large circle or triangle, and is labeled by its rsID. The color intensity of each symbol reflects the extent of LD with the top genotyped SNP, white (*r*^2^=0) through to dark red (*r*^2^=1.0). Genetic recombination rates, estimated using UK10K Genomes Project samples, are shown with a light-blue line. Physical positions are based on NCBI build 37 of the human genome. Also shown are the relative positions of genes and transcripts mapping to the region of association. Genes have been redrawn to show their relative positions; therefore, maps are not to physical scale. The lower panel is the chromatin-state segmentation track (ChromHMM) for lymphoblastoid cells using data from the HapMap ENCODE Project. CNV, copy-number variation.

At 10q26.13, rs35837782 localizes to intron 6 on the gene encoding phospholysine phosphohistidine inorganic pyrophosphate phosphatase (LHPP; Figure 1) with genes *FAM53B* and *METTL10* mapping nearby. The SNP rs4762284 at 12q23.1 maps to intron 1 of the gene encoding the ETS-domain protein (ELK3), with nearby genes including *CDK17* (Figure 1).

To explore the epigenetic profile of association signals at each of the two new risk loci, we used HaploReg and RegulomeDB to examine whether the sentinel SNPs and those in high LD (that is, *r*^2^>0.8 in the 1000 Genomes EUR reference panel) annotate putative transcription factor-binding or enhancer elements (Supplementary Table S9). The SNP rs4762284 resides within a region of open chromatin, common across multiple cell lines, consistent with a regulatory element such as an enhancer or a promoter. To gain further insight into the functional basis of rs35837782 and rs4762284 associations, we examined for an association between SNP genotype and expression of genes mapping within 1 Mb of sentinel SNPs. We made use of publicly available expression data on blood cells, lymphoblastoid cell lines from HapMap3, Geneva and the Multiple Tissue Human Expression Resource pilot data. In blood, rs4762284 genotype was associated with *ELK3* expression at *P*=6.85 × 10^−4^ with the risk allele correlated with reduced expression (Supplementary Table S10).^39^

## Discussion

In this analysis of BCP-ALL, we have identified common variants at 10q26.13 and 12q23.1. It has recently been proposed that many GWAS signals are a consequence of ‘synthetic associations', resulting from the combined effect of one or more rare causal variants rather than simply LD with a common risk variant.^51, 52^ Support for such a model in ALL is provided by the rare high-impact variant rs3731249 in *CDKN2A*^8^ that is in LD with rs77728904. As imputation using UK10K as reference can accurately recover genotypes for variants with MAFs of 0.5%,^12^ the possibility that either 10q26.13 or 12q23.1 associations have a similar genetic basis is highly unlikely.

Given the existence of immunogenetic subtypes of BCP-ALL, it is perhaps not surprising there is variability in the genetic effects on ALL risk by subtype, with 10q21.2 variants influencing hyperdiploid ALL and 10p14 variants influencing non-hyperdiploid/non-*ETV6-RUNX1* disease.^6, 7^ In contrast to the 7p12.2 and 10p12.2 risk variants,^6, 7^ the 10q26.13 and 12q23.1 loci have generic effects on the development of ALL.

Because rs35837782 and rs4762284 localize to *LHPP* and *ELK3*, respectively, it is plausible that the functional basis of these associations are mediated through these genes. ELK3, an ETS-domain transcription factor is an attractive candidate for defining ALL susceptibility *a priori* as it has a role in both B-cell development and IgH gene regulation.^53^ ELK3, which is a member of ETS family of transcription factors, interacting with TCF3 transcription factor 3 (E2A immunoglobulin enhancer-binding factors E12/E47) that is involved in several ALL-specific gene fusions including *TCF3-PBX1*/*t*(1;19)(q23;p13) and *TCF3-HLF*/*t*(17;19)(q23;p13) ALL.^54^ ELK3 is highly expressed primarily at the early stages of B-lymphocyte development with expression declining drastically upon B-cell maturation, correlating with the activity of the enhancer of the immunoglobulin heavy chain.^53^ Hence, genetically determined reduced expression is compatible with B-cell developmental arrest, a hallmark of ALL. In contrast to ELK3 evidence for a role for *LHPP*, which encodes a diphosphatase, in B-cell development or B-cell malignancy is yet to be established.^55^ Although the identified risk SNPs map within regions of active chromatin within B cells and thus have a role in the B-cell *cis*-regulatory network *a priori*, additional laboratory follow-up is required to decipher their functional basis.

In summary, our findings represent a further important step in defining the contribution of inherited genetic variants to the risk of developing ALL. Our current and previous findings are notable because we have defined associations of several regions with susceptibility to ALL, and these regions harbor plausible candidate genes for further investigation. Moreover, they emphasize the role of genetically determined expression of B-cell developmental genes being key players in ALL. Given that there remains significant missing heritability for ALL, future GWAS-based studies in concert with functional analyses are likely to lead to further insights into ALL biology.

## Figures and Tables

**Table 1:** Risk to childhood acute lymphoblastic leukemia at loci 10q26.13 and 12q23.1

*rs35837782 (10q26.13, LHPP)*	*Case genotypes*				*Control genotypes*				*OR*	*95% CI*	*P-value*
	*RAF*	*AA*	*AG*	*GG*	*RAF*	*AA*	*AG*	*GG*			
UK-GWAS	0.67	93	358	373	0.62	745	2510	1945	1.27	(1.14–1.41)	2.04 × 10^−5^
German-GWAS	0.67	93	365	376	0.63	268	952	804	1.19	(1.05–1.36)	0.0072
Meta									1.24	(1.14–1.34)	6.04 × 10^−7^
UK replication 1	0.67	73	211	251	0.62	150	475	405	1.19	(1.03–1.39)	0.022
UK replication 2	0.67	56	265	253	0.61	167	497	391	1.33	(1.14–1.55)	0.0002
German replication	0.65	184	607	621	0.63	204	701	574	1.13	(1.02–1.26)	0.0203
Meta									1.20	(1.11–1.29)	3.66 × 10^−6^
Combined meta									1.21	(1.15–1.28)	1.38 × 10^−11^ (*P*_het_=0.48, *I*^2^=0%)
*rs4762284 (12q23.1, ELK3)*	*RAF*	*AA*	*AT*	*TT*	*RAF*	*AA*	*AT*	*TT*	*OR*	*95% CI*	P*-value*
UK-GWAS	0.33	373	358	94	0.30	2578	2160	462	1.18	(1.05–1.32)	0.0046
German-GWAS	0.33	372	366	95	0.28	1072	773	179	1.30	(1.14–1.49)	1.01 × 10^−4^
Meta									1.23	(1.13–1.34)	2.87 × 10^−6^
UK replication 1	0.31	258	227	52	0.30	497	408	98	1.03	(0.88–1.21)	0.6870
UK replication 2	0.34	232	287	52	0.29	534	434	85	1.30	(1.11–1.53)	0.0008
German replication	0.31	650	625	124	0.28	746	619	109	1.15	(1.02–1.29)	0.0178
Meta									1.16	(1.07–1.25)	3.85 × 10^−4^
Combined meta									1.19	(1.12–1.26)	8.41 × 10^−^^9^ (*P*_het_=0.17, *I*^2^=38%)

Abbreviations: CI, confidence interval; GWAS, genome-wide association study; OR, odds ratio; RAF, risk allele frequency.

Risk allele for rs35837782 is G and risk allele for rs4762284 is T.
