# Supplementary material for: A genome-wide association study identifies risk loci for childhood acute lymphoblastic leukemia at 10q26.13 and 12q23.1
Source: Leukemia. 2016 Nov 11;31(3):573–9. doi: 10.1038/leu.2016.271 (PMC5336191; doi:10.1038/leu.2016.271)
Supplement: Supplementary Information [file leu2016271x1.pdf]

Supplemental Files

A genome-wide association study identifies risk loci for childhood acute lymphoblastic leukemia at 10q26.13 and 12q23.1

Jayaram Vijayakrishnan[1], Rajiv Kumar[2], Marc Y.R. Henrion[1], Anthony V Moorman[4], P Sivaramakrishna Rachakonda[2], Ismail Hosen[2], Miguel Inacio da Silva Filho[2], Amy Holroyd[1], Sara E. Dobbins[1], Rolf Koehler[3], Hauke Thomsen[2], Julie A. Irving[4], James M. Allan[4], Tracy Lightfoot[5], Eve Roman[5], Sally E. Kinsey[6, 7], Eamonn Sheridan[7], Pamela D Thompson[8], Per Hoffmann[9, 16], Markus M. Nöthen[9], Stefanie Heilmann-Heimbach[9], Jöckel Karl-Heinz[11], Mel Greaves[14], Christine J. Harrison[4], Claus R. Bartram[3], Martin Schrappe[13], Martin Stanulla[13], Kari Hemminki[2,15], Richard S. Houlston[1].

[1]Division of Genetics and Epidemiology, The Institute of Cancer Research, Sutton, Surrey, United Kingdom;
[2]Division of Molecular Genetic Epidemiology, German Cancer Research Centre, Heidelberg, Germany;
[3]Institute of Human Genetics, University of Heidelberg, Heidelberg, Germany;
[4]Northern Institute for Cancer Research, Newcastle University, Newcastle upon Tyne, United Kingdom;
[5]Epidemiology and Cancer Statistics Group, Department of Health Sciences, University of York, York, United Kingdom;
[6]Department of Paediatric and Adolescent Haematology and Oncology, Leeds General Infirmary, Leeds, United Kingdom;
[7]Leeds Institute of Molecular Medicine, University of Leeds, Leeds, United Kingdom;
[8]Paediatric and Familial Cancer, Institute of Cancer Sciences, Manchester, United Kingdom;
[9]Institute of Human Genetics, University of Bonn, Bonn, Germany;
[10]German Centre for Neurodegenerative Diseases, Bonn, Germany;
[11]Institute for Medical Informatics, Biometry and Epidemiology, University Hospital Essen, University of Duisburg–Essen, Essen, Germany;
[12]Department of Paediatric Haematology and Oncology, Hannover Medical School, Hannover, Germany;
[13]General Paediatrics, University Hospital Schleswig-Holstein, Kiel, Germany;
[14]Haemato-Oncology Research Unit, Division of Molecular Pathology, Institute of Cancer Research, Sutton, Surrey, United Kingdom;
[15]Center for Primary Health Care Research, Lund University, Malmö, Sweden;
[16]Human Genomics Research Group, Department of Biomedicine, University Hospital Basel, Basel, Switzerland;

Corresponding author: Richard S Houlston; e-mail: richard.houlston@icr.ac.uk; Tel: +44 (0) 208 722 4175; Fax: +44 (0) 208 722 4365

SUPPLEMENTARY FIGURES AND TABLES

Figure S1: Quantile-Quantile (Q-Q) plots of observed and expected χ^2 values of association between all QC filtered SNP genotype and risk of BCP-ALL after imputation with λ_{100} values shown. (a) UK and (b) Germany. The red line represents the null hypothesis of no true association.

Figure S2: Manhattan plot of association P-values. Shown are the genome-wide P values (two sided; –log10P < 10.0) of 11.7 million successfully imputed autosomal SNPs with in a total of 1,658 cases and 7,224 controls from the discovery-phase. Labelled in black are previously identified risk loci and labelled in red are newly identified risk loci. SNPs within 100kb of rs35837782(10q26.13) and rs4762284(12q23.1) are highlighted in green.

Figure S3: Forest plots of ORs for childhood ALL with rs35837782 and rs4762284 genotype. ORs for all BCP-ALL (A), Hyperdiploid (B), ETV6-RUNX1 (C), Other:Non-Hyperdiploid/ETV6-RUNX1 ALL (D).OR: odds ratio; CI: confidence intervals Phet :P -value for heterogeneity. I2 : variation attributable to heterogenity.

Figure S4: Kaplan-Meir curves of patient survivorship in BCP-ALL patients. (a-f) Stratified by rs35837782 (10p14) and rs4762284 (g-l) genotype. EFS: Event Free Survivorship; SR: standard risk; HR: higher risk; MR: medium risk; CI: cumulative incidence.

Figure S5: Population distribution of childhood ALL relative scores ordered by genetic risk (risk is relative to population median risk). The blue line plots the distribution of RR across the population;the red lines correspond to 1st, 10th 90th and 99th centiles. The RR figures presented in black are the average in the (i) highest 10 and (ii) top 1 centile of genetic risk.

Table S1: Summary statistics for novel variants showing an association with BCP-ALL risk in the GWAS meta-analysis after QC. a: Shown are discovery association P values for individual studies and from meta-analysis of GWAS datasets. a. P-values under a fixed effects model. b. P-values under a random effects model c:OR Odds ratios derived with respect to the risk allele.RAF: risk allele frequency in discovery cases. *SNPs which were genome-wide significant after replication genotyping.# rs6933843 was used a proxy to rs6569588 which was the top ranking SNP in that candidate loci.

Table S2: Imputation quality scores and concordance between directly sequenced and imputed genotype. (a) SNPs which were genome-wide significant after replication; (b) SNPs which were not genome-wide significant after replication. AA, major homozygote; AB, heterozygote; BB, minor homozygote; Is, Information score giving an indication of imputation quality. r^2 indicates Pearson product-moment correlation coefficient between imputed and sequenced genotype.

Table S3: Replication series for BCP-ALL GWAS. Candidate SNPs genotyped in UK Replication 1 and German Replication series, SNPs nominally significant (i.e. P<0.05) in both series combined were genotyped in a third UK Replication series 2 to identify novel risk

factors. CHR: Chromosome, POS: Position in Hg19 build, A1/A2: Alleles, P-value obtained under a logistic regression model.

Table S4: Relationship between SNP genotype and ALL subtype. RAF: Risk Allele Frequency, OR: Odds Ratio, P-value obtained from logistic regression.

Table S5: Conditional analysis of SNPs significant in 9p21.3 region and newly claimed independent SNP rs77728904. P-values obtained from a meta-analysis of B cell ALL UK and German GWAS series after imputation with UK10k and 1KG reference panels. rs3731249 is the recently described low frequency variant by Vijayakrishnan et al. P-values* obtained through meta-analysis under a fixed model.

Table S6: Relationship between genotype and specific tumor karyotype. P-values are given for case only analysis, along with corresponding numbers (n) and risk allele frequencies (RAF) in parenthesis.*status for selected chromosome anomalies.[$]CDKN2A deletion status available for combined UK replication 1 and 2 data sets.

Table S7. Hazard ratios for an event for rs35837782 and rs4762284 by genotype. a.in comparison to heterozygous patients.

Table S8. Multivariate hazard ratios for an event for rs35837782 and rs4762284 by genotype.

Table S9: Epigenetic annotation of genome-wide significant SNPs. Data are shown for query SNPs and their proxies ($r2 > 0.2$ in 1000 Genomes EUR Phase 1 data) demonstrating evidence of histone marks, DNase hypersensitivity sites or transcription factor occupancy in RegulomeDB analysis. Also indicated are genomic evolutionary rate profiling (GERP) scores and combined annotation dependent depletion (CADD) scores. Chr, chromosome; DNAse HS, DNAse hypersensitivity; Fre, frequency; GERP, Genomic Evolutionary Rate Profiling; LD, linkage disequilibrium; Pos, position; CADD, combined annotation dependent depletion. RegulomeDB scores: 2b, TF binding + any motif + Dnase Footprint + Dnase peak; 4, TF binding + DNAse peak; 5, TF binding or Dnase peak; 6, other binding or Dnase peak.

Table S10: eQTL analysis in LCL and blood tissues for rs4762284 using the eQTL blood browser online resources. Genes within 1 Mb of query SNP were assessed for association. *FDR: False discovery rate values shown are from 'probe level' rates as described by Westra H. et al, Nat.Gen (2012).

Table S11: Oligonucleotides used for KASPAR genotyping and Sanger sequencing.

a.

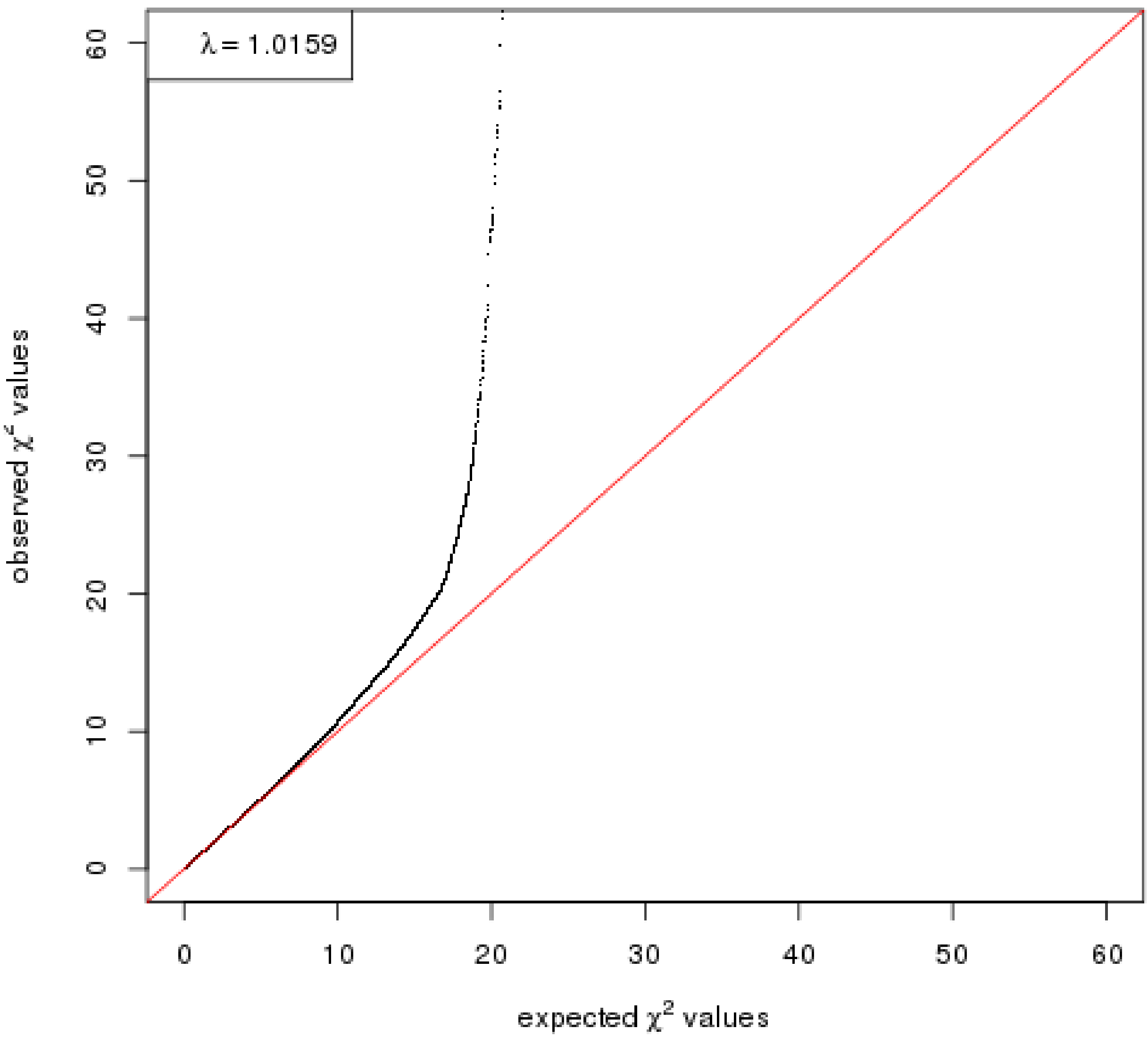
λ = 1.0159
observed χ² values
expected χ² values
0
10
20
30
40
50
60

b.

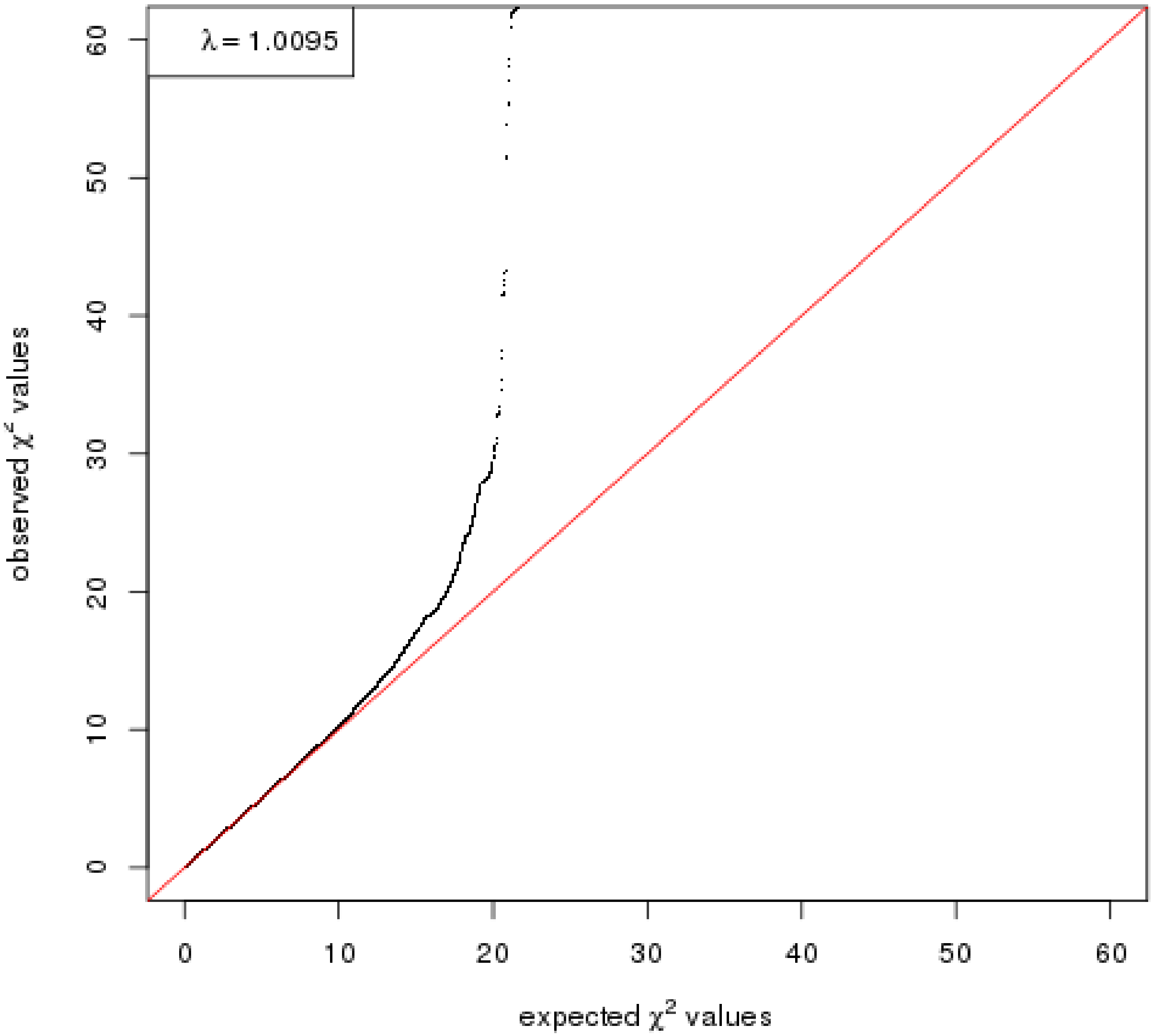

λ = 1.0095
observed χ² values
expected χ² values
0
10
20
30
40
50
60

Figure S1: Quantile-Quantile (Q-Q) plots of observed and expected χ^2 values of association between all QC filtered SNP genotype and risk of BCP-ALL after imputation with λ_{100} values shown. (a) UK and (b) Germany. The red line represents the null hypothesis of no true association.

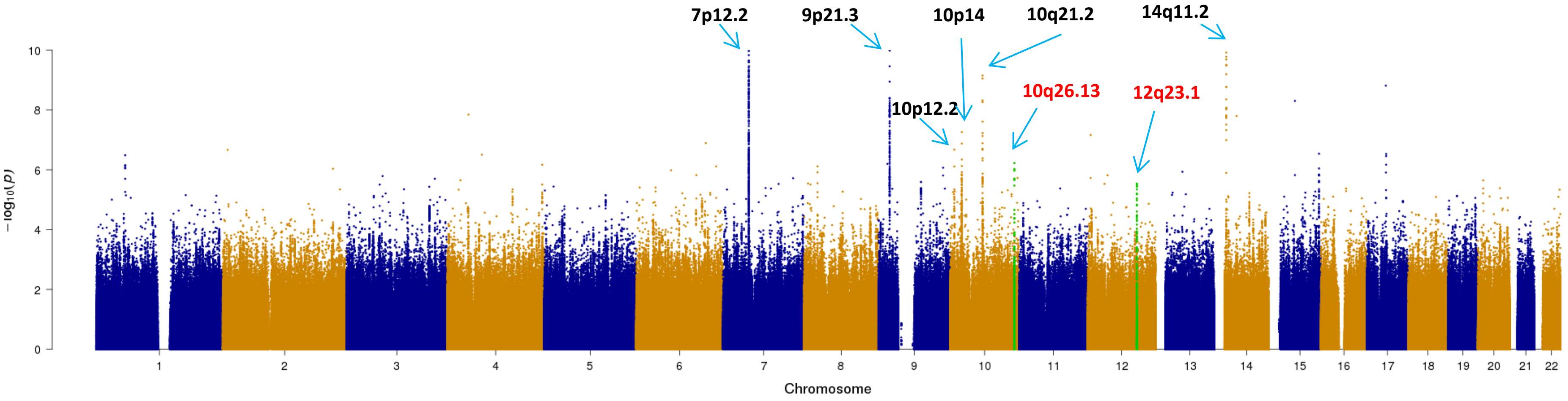

Figure S2: Manhattan plot of association P-values. Shown are the genome-wide *P* values (two sided; $-\log_{10}P < 10.0$) of 11.7 million successfully imputed autosomal SNPs with in a total of 1,658 cases and 7,224 controls from the discovery-phase. Labelled in black are previously identified risk loci and labelled in red are newly identified risk loci. SNPs within 100kb of rs35837782(10q26.13) and rs4762284(12q23.1) are highlighted in green.

A

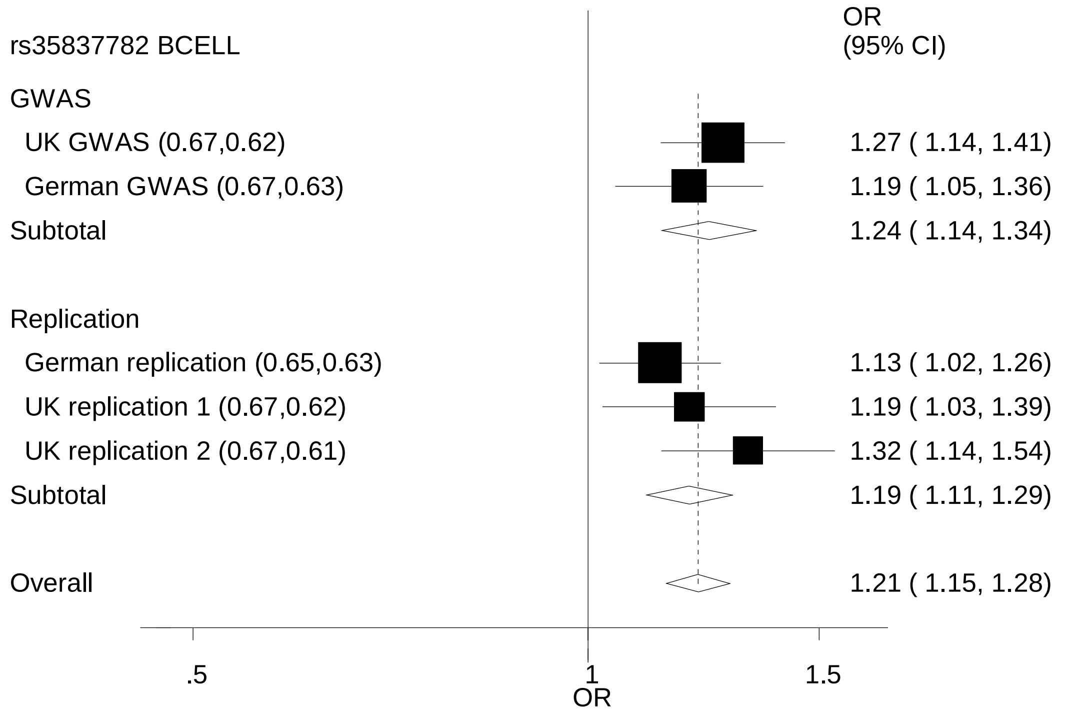

P-value =1.38X10^{-11}; P_{het} = 0.48,, I^2 = 0%

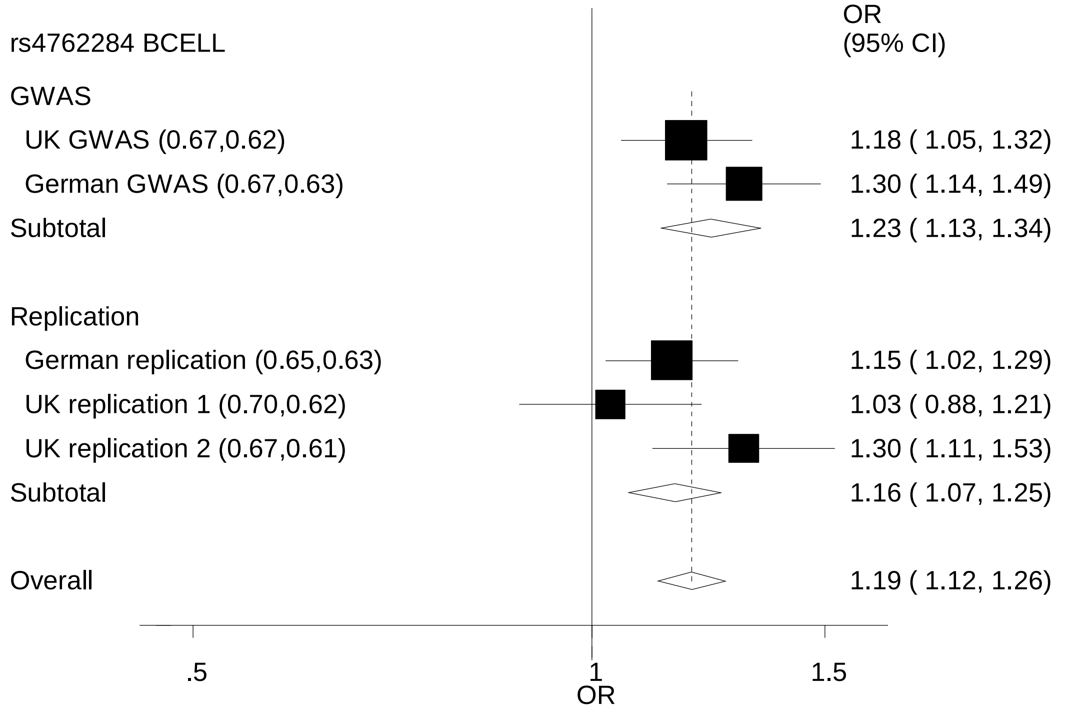

P-value =8.41X10^{-9}; P_{het} = 0.17, I^2 = 38%

B

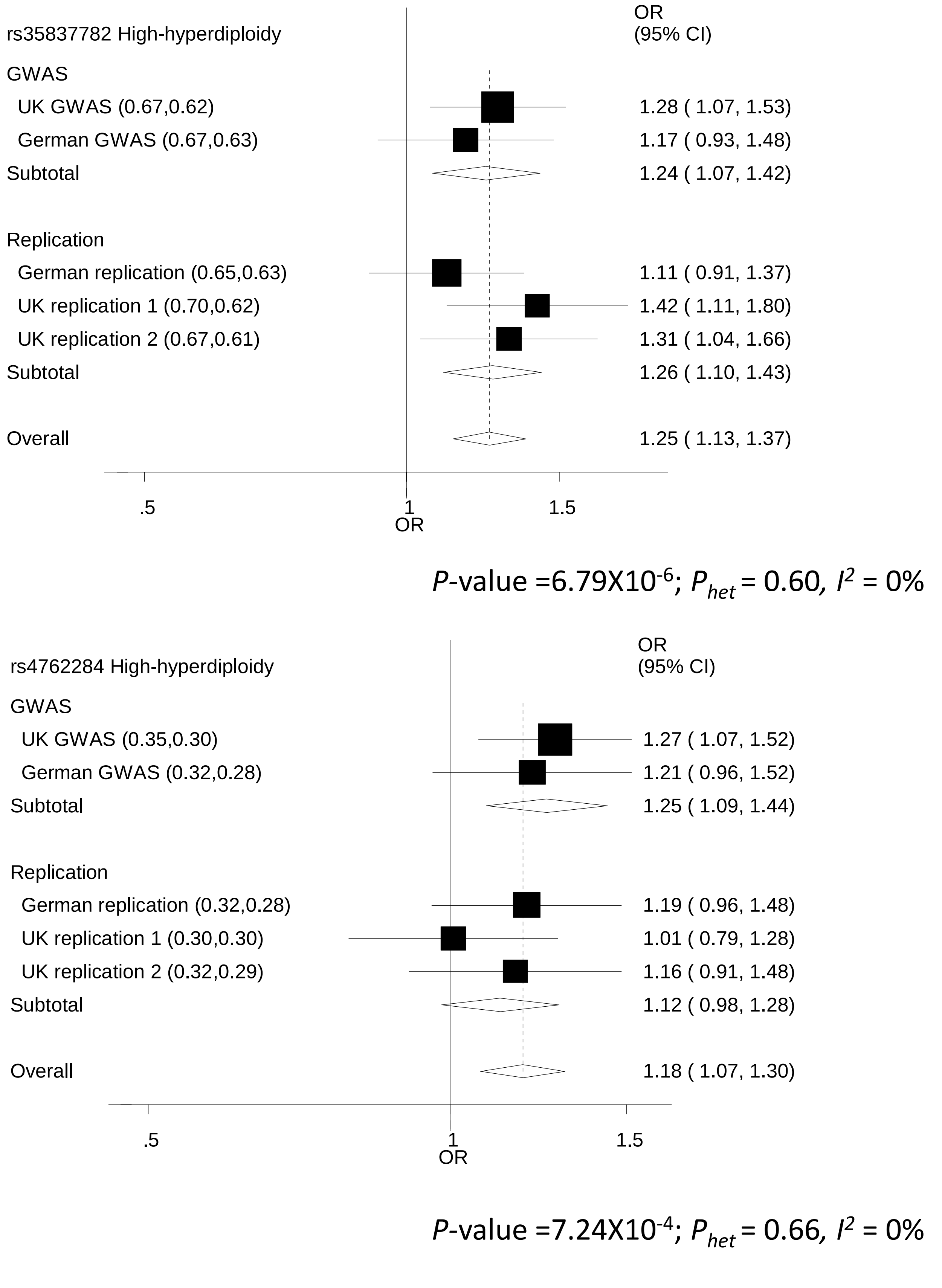
rs35837782 High-hyperdiploidy
OR
(95% CI)
GWAS
UK GWAS (0.67,0.62)
1.28 (1.07, 1.53)
German GWAS (0.67,0.63)
1.17 (0.93, 1.48)
Subtotal
1.24 (1.07, 1.42)
Replication
German replication (0.65,0.63)
1.11 (0.91, 1.37)
UK replication 1 (0.70,0.62)
1.42 (1.11, 1.80)
UK replication 2 (0.67,0.61)
1.31 (1.04, 1.66)
Subtotal
1.26 (1.10, 1.43)
Overall
1.25 (1.13, 1.37)
.5
1
1.5
OR
P-value =6.79X10^{-6}; P_{het} = 0.60, I^2 = 0%
rs4762284 High-hyperdiploidy
OR
(95% CI)
GWAS
UK GWAS (0.35,0.30)
1.27 (1.07, 1.52)
German GWAS (0.32,0.28)
1.21 (0.96, 1.52)
Subtotal
1.25 (1.09, 1.44)
Replication
German replication (0.32,0.28)
1.19 (0.96, 1.48)
UK replication 1 (0.30,0.30)
1.01 (0.79, 1.28)
UK replication 2 (0.32,0.29)
1.16 (0.91, 1.48)
Subtotal
1.12 (0.98, 1.28)
Overall
1.18 (1.07, 1.30)
.5
1
1.5
OR
P-value =7.24X10^{-4}; P_{het} = 0.66, I^2 = 0%

C

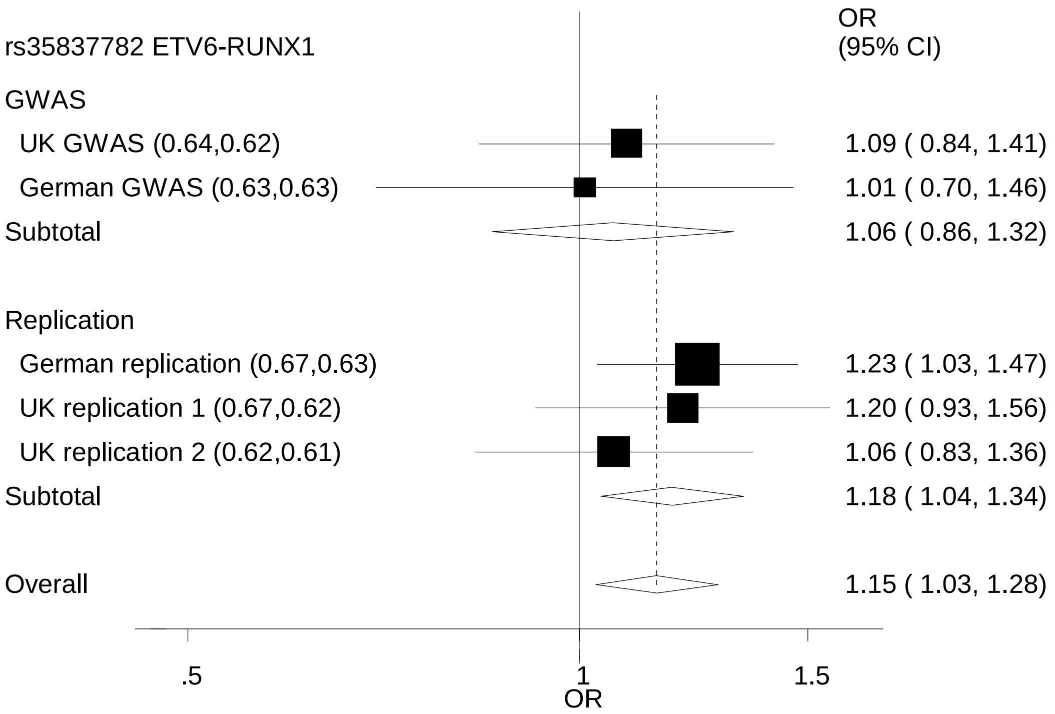

P-value =0.01; P_{het} = 0.76, I^2 = 0%

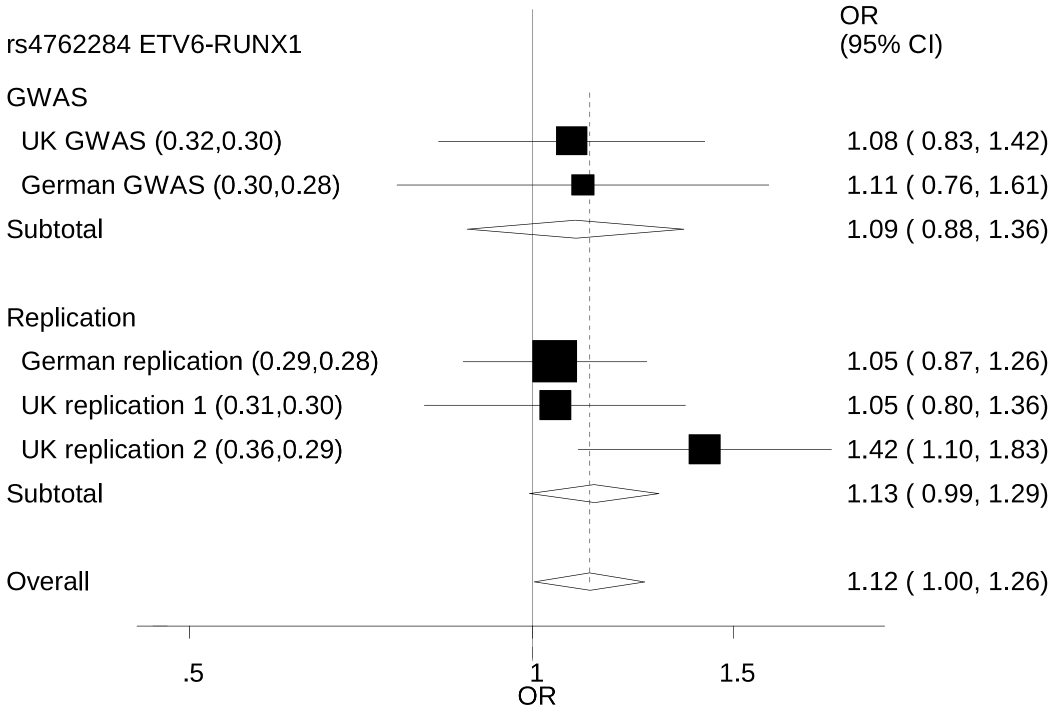

P-value =0.05; P_{het} = 0.40, I^2 = 1.5%

D

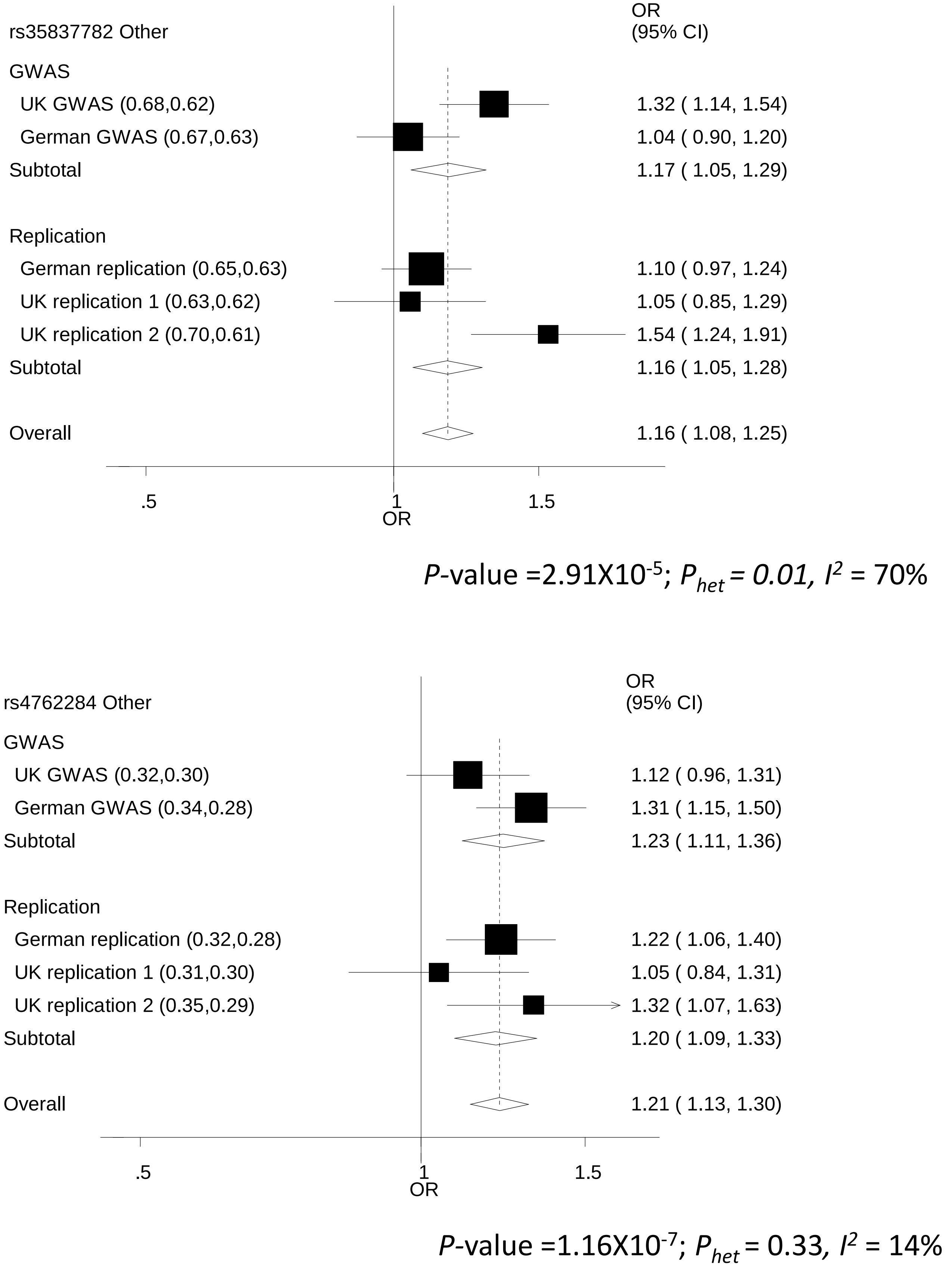

rs35837782 Other
OR
(95% CI)
GWAS
UK GWAS (0.68,0.62)
1.32 (1.14, 1.54)
German GWAS (0.67,0.63)
1.04 (0.90, 1.20)
Subtotal
1.17 (1.05, 1.29)
Replication
German replication (0.65,0.63)
1.10 (0.97, 1.24)
UK replication 1 (0.63,0.62)
1.05 (0.85, 1.29)
UK replication 2 (0.70,0.61)
1.54 (1.24, 1.91)
Subtotal
1.16 (1.05, 1.28)
Overall
1.16 (1.08, 1.25)
.5
1
1.5
OR
P-value =2.91X10-5; Phet = 0.01, I2 = 70%
rs4762284 Other
OR
(95% CI)
GWAS
UK GWAS (0.32,0.30)
1.12 (0.96, 1.31)
German GWAS (0.34,0.28)
1.31 (1.15, 1.50)
Subtotal
1.23 (1.11, 1.36)
Replication
German replication (0.32,0.28)
1.22 (1.06, 1.40)
UK replication 1 (0.31,0.30)
1.05 (0.84, 1.31)
UK replication 2 (0.35,0.29)
1.32 (1.07, 1.63)
Subtotal
1.20 (1.09, 1.33)
Overall
1.21 (1.13, 1.30)
.5
1
1.5
OR
P-value =1.16X10-7; Phet = 0.33, I2 = 14%

Figure S3: **Forest plots of ORs for childhood ALL with rs35837782 and rs4762284 genotype.** ORs for all BCP-ALL (A), Hyperdiploid (B), ETV6-RUNX1 (C), Other:Non-Hyperdiploid/ETV6-RUNX1 ALL (D).OR: odds ratio; CI: confidence intervals P_{het} :P -value for heterogeneity. I^2 : variation attributable to heterogenity.

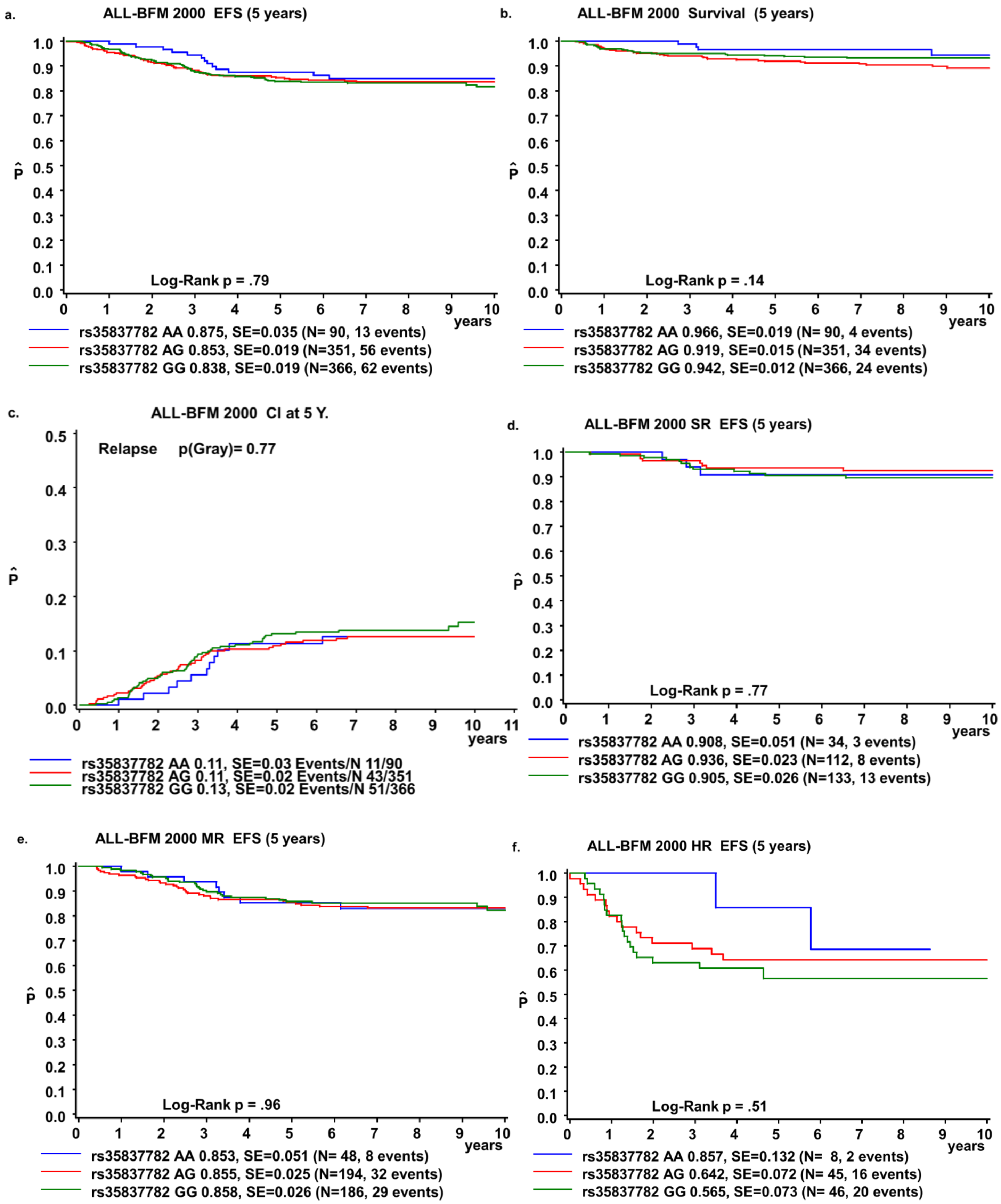
a.
ALL-BFM 2000 EFS (5 years)
Log-Rank p = .79
rs35837782 AA 0.875, SE=0.035 (N= 90, 13 events)
rs35837782 AG 0.853, SE=0.019 (N=351, 56 events)
rs35837782 GG 0.838, SE=0.019 (N=366, 62 events)
b.
ALL-BFM 2000 Survival (5 years)
Log-Rank p = .14
rs35837782 AA 0.966, SE=0.019 (N= 90, 4 events)
rs35837782 AG 0.919, SE=0.015 (N=351, 34 events)
rs35837782 GG 0.942, SE=0.012 (N=366, 24 events)
c.
ALL-BFM 2000 CI at 5 Y.
Relapse p(Gray)= 0.77
rs35837782 AA 0.11, SE=0.03 Events/N 11/90
rs35837782 AG 0.11, SE=0.02 Events/N 43/351
rs35837782 GG 0.13, SE=0.02 Events/N 51/366
d.
ALL-BFM 2000 SR EFS (5 years)
Log-Rank p = .77
rs35837782 AA 0.908, SE=0.051 (N= 34, 3 events)
rs35837782 AG 0.936, SE=0.023 (N=112, 8 events)
rs35837782 GG 0.905, SE=0.026 (N=133, 13 events)
e.
ALL-BFM 2000 MR EFS (5 years)
Log-Rank p = .96
rs35837782 AA 0.853, SE=0.051 (N= 48, 8 events)
rs35837782 AG 0.855, SE=0.025 (N=194, 32 events)
rs35837782 GG 0.858, SE=0.026 (N=186, 29 events)
f.
ALL-BFM 2000 HR EFS (5 years)
Log-Rank p = .51
rs35837782 AA 0.857, SE=0.132 (N= 8, 2 events)
rs35837782 AG 0.642, SE=0.072 (N= 45, 16 events)
rs35837782 GG 0.565, SE=0.073 (N= 46, 20 events)
P̂
years

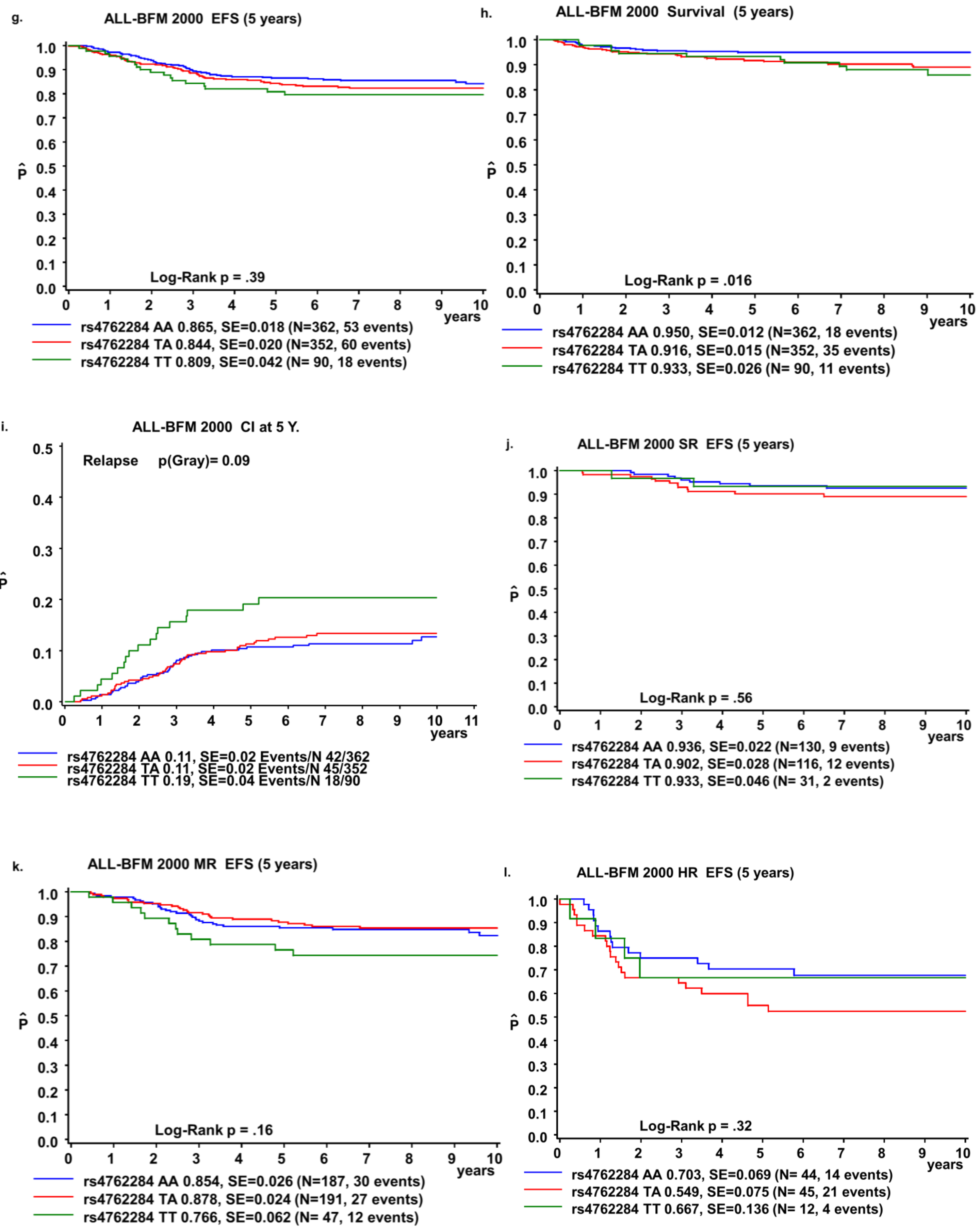
g.
ALL-BFM 2000 EFS (5 years)
Log-Rank p = .39
years
rs4762284 AA 0.865, SE=0.018 (N=362, 53 events)
rs4762284 TA 0.844, SE=0.020 (N=352, 60 events)
rs4762284 TT 0.809, SE=0.042 (N= 90, 18 events)
h.
ALL-BFM 2000 Survival (5 years)
Log-Rank p = .016
years
rs4762284 AA 0.950, SE=0.012 (N=362, 18 events)
rs4762284 TA 0.916, SE=0.015 (N=352, 35 events)
rs4762284 TT 0.933, SE=0.026 (N= 90, 11 events)
i.
ALL-BFM 2000 CI at 5 Y.
Relapse p(Gray)= 0.09
years
rs4762284 AA 0.11, SE=0.02 Events/N 42/362
rs4762284 TA 0.11, SE=0.02 Events/N 45/352
rs4762284 TT 0.19, SE=0.04 Events/N 18/90
j.
ALL-BFM 2000 SR EFS (5 years)
Log-Rank p = .56
years
rs4762284 AA 0.936, SE=0.022 (N=130, 9 events)
rs4762284 TA 0.902, SE=0.028 (N=116, 12 events)
rs4762284 TT 0.933, SE=0.046 (N= 31, 2 events)
k.
ALL-BFM 2000 MR EFS (5 years)
Log-Rank p = .16
years
rs4762284 AA 0.854, SE=0.026 (N=187, 30 events)
rs4762284 TA 0.878, SE=0.024 (N=191, 27 events)
rs4762284 TT 0.766, SE=0.062 (N= 47, 12 events)
l.
ALL-BFM 2000 HR EFS (5 years)
Log-Rank p = .32
years
rs4762284 AA 0.703, SE=0.069 (N= 44, 14 events)
rs4762284 TA 0.549, SE=0.075 (N= 45, 21 events)
rs4762284 TT 0.667, SE=0.136 (N= 12, 4 events)

Figure S4: Kaplan-Meir curves of patient survivorship in BCP-ALL patients. (a-f) Stratified by rs35837782 (10p14) and rs4762284 (g-l) genotype. EFS: Event Free Survivorship; SR: standard risk; HR: higher risk; MR: medium risk; CI: cumulative incidence.

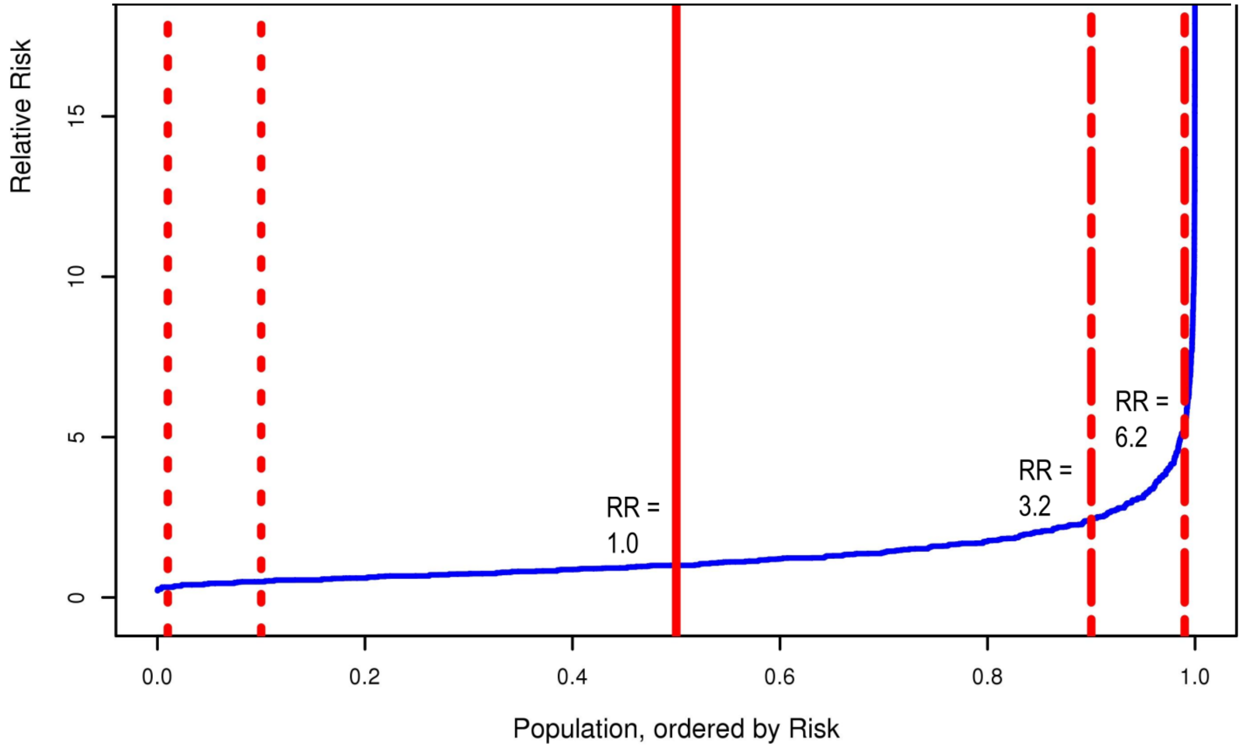

Figure S5: Population distribution of childhood ALL relative risk scores ordered by genetic risk (risk is relative to population median risk). The blue line plots the distribution of RR across the population; the red lines correspond to 1st, 10th, 50th, 90th and 99th centiles. The RR figures presented in black are the average in the (i) highest 10 and (ii) top 1 centile of genetic risk.

										UK series				German series				
											RAF				RAF			
**SNP**	**Chr**	**Position**	**A1**	**A2**	P^{a}	P^{b}	**OR**^{c}	**Q**	**I2**	**INFO**	**Case**	**Control**	***P* value**	**INFO**	**Case**	**Control**	***P* value**	**Gene/Feature**
rs12751998	1	57193872	T	G	3.26X10^{-07}	3.26X10^{-07}	1.97	0.32	0	0.96	0.04	0.02	3.48X10^{-06}	0.97	0.03	0.02	1.84X10^{-02}	C1orf168
rs1869595	1	110746260	G	A	3.88X10^{-05}	3.88X10^{-05}	0.85	0.63	0	1	0.47	0.51	4.88X10^{-04}	0.97	0.46	0.5	2.53X10^{-02}	Intergenic
rs890579	2	179263287	C	T	5.41X10^{-05}	5.41X10^{-05}	0.85	0.98	0	0.99	0.37	0.41	1.79X10^{-03}	1	0.37	0.4	1.05X10^{-02}	OSBPL6
rs74952692	3	162926039	C	T	3.23X10^{-05}	3.23X10^{-05}	1.4	0.45	0	0.99	0.09	0.06	2.22X10^{-04}	1	0.08	0.06	4.01X10^{-02}	LINC1192
rs16852911	4	41205654	A	T	2.03X10^{-05}	2.03X10^{-05}	1.48	0.97	0	0.99	0.06	0.05	1.46X10^{-03}	1	0.06	0.05	4.59X10^{-03}	APBB2
rs145666495	4	186977423	G	T	6.75X10^{-07}	6.75X10^{-07}	2.54	0.38	0	0.81	0.03	0.02	2.38X10^{-04}	0.74	0.02	0.01	5.48X10^{-04}	Intergenic
rs3748079	6	33588147	C	T	5.05X10^{-05}	5.05X10^{-05}	1.22	0.61	0	1	0.22	0.2	7.88X10^{-03}	1	0.28	0.24	1.91X10^{-03}	Intergenic
rs6933843[#]	6	129551759	T	G	1.08X10^{-05}	1.08X10^{-05}	2.18	0.58	0	0.9	0.03	0.02	1.27X10^{-04}	0.97	0.02	0.01	2.55X10^{-02}	LAMA2
rs3750131	7	156754981	T	C	7.43X10^{-06}	7.43X10^{-06}	1.25	0.83	0	1	0.25	0.21	3.39X10^{-04}	1	0.23	0.2	6.96X10^{-03}	NOM1
rs3935421	8	130203139	A	G	6.51X10^{-05}	6.51X10^{-05}	1.28	0.86	0	1	0.14	0.12	1.99X10^{-03}	0.98	0.16	0.12	1.13X10^{-02}	Intergenic
rs62579834	9	83747824	C	A	2.54X10^{-06}	2.54X10^{-06}	0.58	0.7	0	0.94	0.02	0.03	1.39X10^{-04}	0.95	0.02	0.04	5.29X10^{-03}	Intergenic
**rs35837782***	**10**	**126293309**	**A**	**G**	**5.96X10^{-07}**	**5.96X10^{-07}**	**1.24**	**0.5**	**0**	**0.98**	**0.33**	**0.38**	**2.04X10^{-05}**	**0.98**	**0.33**	**0.37**	**7.19X10^{-03}**	**LHPP**
rs16937230	11	8264350	T	C	1.30X10^{-05}	8.36X10^{-05}	0.66	0.27	17	0.91	0.03	0.06	5.43X10^{-05}	1	0.04	0.05	4.75X10^{-02}	LMO1
rs1001205	11	119292712	T	C	7.48X10^{-05}	7.48X10^{-05}	1.18	0.8	0	0.99	0.38	0.41	4.26X10^{-03}	1	0.38	0.43	5.90X10^{-03}	THY1
rs1419981	12	7756706	A	G	5.55X10^{-05}	5.55X10^{-05}	1.38	0.36	0	0.98	0.08	0.06	3.56X10^{-04}	1	0.09	0.07	3.73X10^{-02}	Intergenic
**rs4762284***	**12**	**96612762**	**A**	**T**	**2.90X10^{-06}**	**2.69X10^{-05}**	**1.23**	**0.26**	**20**	**0.98**	**0.33**	**0.3**	**4.63X10^{-03}**	**0.97**	**0.33**	**0.28**	**1.01X10^{-04}**	**ELK3**
rs7152152	14	68906285	C	T	1.87X10^{-05}	2.18X10^{-03}	0.82	0.16	51	0.99	0.28	0.26	1.80X10^{-02}	1	0.29	0.24	1.23X10^{-04}	RAD51B
rs10148274	14	94087775	C	A	5.86X10^{-05}	5.86X10^{-05}	0.83	0.39	0	0.95	0.32	0.3	1.23X10^{-02}	1	0.32	0.27	1.12X10^{-03}	UNC79
rs4448919	15	98663026	G	A	9.01X10^{-07}	9.01X10^{-07}	1.23	0.9	0	0.97	0.47	0.42	1.36X10^{-04}	0.97	0.48	0.44	1.96X10^{-03}	Intergenic
rs10411772	19	54048951	A	C	8.71X10^{-06}	8.02X10^{-04}	1.44	0.18	45	0.89	0.09	0.07	1.20X10^{-02}	1	0.08	0.05	9.30X10^{-05}	ZNF331

Table S1: Summary statistics for novel variants showing an association with BCP-ALL risk in the GWAS meta-analysis after QC. a: Shown are discovery association P values for individual studies and from meta-analysis of GWAS datasets. a. *P*-values under a fixed effects model. b. *P*-values under a random effects model c:OR Odds ratios derived with respect to the risk allele.RAF: risk allele frequency in discovery cases. *SNPs which were genome-wide significant after replication genotyping.[#] rs6933843 was used a proxy to rs6569588 which was the top ranking SNP in that candidate loci.

SNP	Alleles	Concordance	r2
rs1001205	AA	54/54	1.0
	Aa	76/76	
	aa	16/16	
rs890579	AA	52/52	1.0
	Aa	69/69	
	aa	27/27	
rs1419881	AA	117/118	0.999
	Aa	22/24	
	Aa	2/2	
**rs4762284**[a]	AA	78/78	1.0
	Aa	51/51	
	aa	17/17	
rs6933843	AA	0/0	1.0
	Aa	5/5	
	aa	139/139	
rs10148274	AA	64/65	0.999
	Aa	56/59	
	Aa	12/12	
rs1041172	AA	116/116	0.999
	Aa	19/20	
	aa	0/0	
rs16937230	AA	0/0	0.999
	Aa	10/12	
	aa	134/135	
**rs35837782**[a]	AA	17/18	0.999
	Aa	62/62	
	Aa	63/64	
rs74952692	AA	126/126	1.0
	Aa	22/22	
	Aa	0/0	
rs16852911	AA	134/134	0.999
	Aa	12/13	
	aa	2/2	
rs12751998	AA	0/0	1.0
	Aa	10/10	
	aa	137/137	
rs4448919	AA	34/35	0.999
	Aa	67/68	
	Aa	34/36	
rs145666495	AA	141/142	1.0
	Aa	5/5	
	aa	0/0	
rs7152152	AA	11/11	0.999
	Aa	54/55	
	Aa	80/80	
rs62579834	AA	1/1	1.0
	Aa	3/3	
	Aa	144/144	

Table S2: Imputation quality scores and concordance between directly sequenced and imputed genotype. (a) SNPs which were genome-wide significant after replication. AA, major homozygote; AB, heterozygote; BB, minor homozygote; Is, Information score giving an indication of imputation quality. r^2 indicates Pearson product-moment correlation coefficient between imputed and sequenced genotype.

**UK Replication 1**						**Cases**				**Controls**			
**SNP**	**CHR**	**POS**	**A1**	**A2**	**AA**	**AB**	**BB**	**RAF**	**AA**	**AB**	**BB**	**RAF**	***P*-value**
rs12751998	1	57193872	T	G	0	22	351	0.97	1	59	961	0.97	0.77
rs1869595	1	110746260	G	A	139	250	148	0.51	266	514	256	0.50	0.49
rs890579	2	179263287	C	T	171	244	115	0.45	379	489	149	0.39	0.001
rs16852911	4	41205654	A	T	3	61	478	0.94	0	95	952	0.95	0.06
rs3748079	6	33588147	C	T	321	185	31	0.23	653	328	36	0.20	0.03
rs3750131	7	156754981	T	C	26	186	332	0.78	50	323	631	0.79	0.60
rs62579834	9	83747824	C	A	0	25	517	0.98	3	81	939	0.96	0.02
**rs35837782**	**10**	**126293309**	**A**	**G**	**73**	**211**	**251**	0.67	**150**	**475**	**405**	**0.62**	**0.02**
rs1001205	11	119292712	T	C	210	246	81	0.38	350	496	175	0.41	0.06
**rs4762284**	**12**	**96612762**	**A**	**T**	**258**	**227**	**52**	0.31	**497**	**408**	**98**	**0.30**	**0.69**
rs7152152	14	68906285	C	T	31	210	294	0.75	69	359	591	0.76	0.53
rs10148274	14	94087775	C	A	245	232	60	0.33	494	424	94	0.30	0.15
rs4448919	15	98663026	G	A	120	268	148	0.53	184	504	325	0.57	0.02
rs10411772	19	54048951	A	C	448	80	7	0.09	880	141	2	0.07	0.09

**German Replication**						**Cases**				**Controls**			
**SNP**	**CHR**	**POS**	**A1**	**A2**	**AA**	**AB**	**BB**	**RAF**	**AA**	**AB**	**BB**	**RAF**	***P*-value**
rs12751998	1	57193872	T	G	3	70	1315	0.97	2	61	1424	0.98	0.19
rs1869595	1	110746260	G	A	345	703	428	0.53	315	704	461	0.55	0.11
rs890579	2	179263287	C	T	208	667	527	0.61	236	718	524	0.60	0.20
rs16852911	4	41205654	A	T	1229	151	6	0.06	1351	131	7	0.05	0.10
rs3748079	6	33588147	C	T	746	540	113	0.27	821	590	83	0.25	0.07
rs3750131	7	156754981	T	C	87	494	806	0.76	62	498	919	0.79	0.01
rs62579834	9	83747824	C	A	1	90	1308	0.97	3	98	1386	0.97	0.66
**rs35837782**	**10**	**126293309**	**A**	**G**	**184**	**607**	**621**	**0.65**	**204**	**701**	**574**	**0.63**	**0.02**
rs1001205	11	119292712	T	C	533	660	207	0.38	543	708	235	0.40	0.32
**rs4762284**	**12**	**96612762**	**A**	**T**	**650**	**625**	**124**	**0.31**	**746**	**619**	**109**	**0.28**	**0.02**
rs7152152	14	68906285	C	T	118	525	757	0.73	112	549	818	0.74	0.38
rs10148274	14	94087775	C	A	666	587	138	0.31	752	593	140	0.29	0.19
rs4448919	15	98663026	G	A	302	647	434	0.55	296	749	412	0.54	0.55
rs10411772	19	54048951	A	C	1197	187	9	0.07	1288	192	7	0.07	0.53

UK Replication 2					Cases				Controls				
**SNP**	**CHR**	**POS**	**A1**	**A2**	**AA**	**AB**	**BB**	**RAF**	**AA**	**AB**	**BB**	**RAF**	***P*-value**
rs890579	2	179263287	C	T	188	274	112	0.43	401	515	140	0.38	0.001
rs3748079	6	33588147	C	T	358	197	20	0.21	665	350	45	0.21	0.92
rs3750131	7	156754981	T	C	26	185	364	0.79	39	343	678	0.80	0.61
**rs35837782**	**10**	**126293309**	**A**	**G**	**56**	**265**	**253**	**0.67**	**167**	**497**	**391**	**0.61**	**0.0002**
**rs4762284**	**12**	**96612762**	**A**	**T**	**232**	**287**	**52**	**0.34**	**534**	**434**	**85**	**0.29**	**0.0008**

Table S3: Replication series for BCP-ALL GWAS. Candidate SNPs genotyped in UK Replication 1 and German Replication series, SNPs nominally significant (i.e. $P<0.05$) in both series combined were genotyped in a third UK Replication series 2 to identify novel risk factors. CHR: Chromosome, POS: Position in Hg19 build, A1/A2: Alleles, *P*-value obtained under a logistic regression model.

**Hyperdiploid**	**Case genotypes**					**Control genotypes**							
**rs35837782 (10q26.13, *LHPP*)**	**RAF[a](G)**	**AA**	**AG**	**GG**	**Total**	**RAF(G)**	**AA**	**AG**	**GG**	**Total**	**OR[b]**	**95% CI[c]**	***P*-value**
UK GWAS	0.67	31	128	129	289	0.62	745	2510	1945	5200	1.28	(1.07-1.53)	0.007
German GWAS	0.67	15	87	74	176	0.63	268	952	804	2024	1.17	(0.93-1.48)	0.147
German replication	0.65	32	97	101	230	0.63	204	701	574	1479	1.11	(0.91-1.37)	0.304
UK replication 1	0.7	22	62	95	179	0.62	150	475	405	1030	1.42	(1.11-1.80)	0.005
UK replication 2	0.67	18	84	80	182	0.61	167	500	395	1062	1.31	(1.04-1.66)	0.023
**Combined**	**0.67**	**118**	**459**	**479**	**1056**	**0.62**	**1534**	**5139**	**4123**	**10795**	**1.25**	**(1.13-1.37)**	**6.79×10^{-6}**
												(P_{het} = 0.60, I^2= 0%)	
**rs4762284 (12q23.1,*ELK3*)**	**RAF(T)**	**AA**	**AT**	**TT**	**Total**	**RAF(T)**	**AA**	**AT**	**TT**	**Total**	**OR**	**95% CI**	***P*-value**
UK GWAS	0.35	121	134	34	289	0.3	2578	2160	462	5200	1.27	(1.07-1.52)	0.006
German GWAS	0.32	82	75	19	176	0.28	1072	773	179	2024	1.21	(0.96-1.52)	0.084
German replication	0.32	100	109	18	227	0.28	746	619	109	1474	1.19	(0.96-1.48)	0.113
UK replication 1	0.3	88	75	17	180	0.3	497	408	98	1003	1.01	(0.79-1.28)	0.95
UK replication 2	0.32	76	96	10	182	0.29	534	434	85	1053	1.16	(0.94-1.48)	0.208
**Combined**	**0.33**	**466**	**489**	**98**	**1054**	**0.29**	**5428**	**4394**	**932**	**10754**	**1.18**	**(1.07-1.30)**	**7.24×10^{-4}**
												(P_{het}= 0.66, I^2= 0%)	

ETV6/RUNX1	**Case genotypes**					**Control genotypes**							
**rs35837782 (10q26.13, *LHPP*)**	**RAF[a](G)**	**AA**	**AG**	**GG**	**Total**	**RAF(G)**	**AA**	**AG**	**GG**	**Total**	**OR[b]**	**95% CI[c]**	***P*-value**
UK GWAS	0.64	17	58	51	126	0.62	745	2510	1945	5200	1.09	(0.84-1.41)	0.498
German GWAS	0.63	9	28	26	63	0.63	268	952	804	2024	1.01	(0.70-1.46)	0.842
German replication	0.67	36	150	153	339	0.63	204	701	574	1479	1.23	(1.03-1.47)	0.02
UK replication 1	0.67	16	62	63	141	0.62	150	475	405	1030	1.20	(0.93-1.56)	0.166
UK replication 2	0.62	24	66	61	151	0.61	167	500	395	1062	1.06	(0.83-1.36)	0.617
**Combined**	**0.65**	**102**	**365**	**354**	**820**	**0.62**	**1534**	**5139**	**4123**	**10795**	**1.15**	**(1.03-1.28)**	**0.01**
												(P_{het} = 0.76, I^2= 0%)	

rs4762284 (12q23.1,*ELK3*)	RAF(T)	AA	AT	TT	Total	RAF(T)	AA	AT	TT	Total	OR	95% CI	*P*-value
UK GWAS	0.32	60	53	13	126	0.3	2578	2160	462	5200	1.08	(0.83-1.42)	0.520
German GWAS	0.3	33	22	8	63	0.28	1072	773	179	2024	1.11	(0.76-1.61)	0.624
German replication	0.29	169	137	30	336	0.28	746	619	109	1474	0.97	(0.80-1.16)	0.628
UK replication 1	0.31	68	61	14	143	0.3	497	408	98	1003	1.12	(0.85-1.46)	0.732
UK replication 2	0.36	58	75	17	150	0.29	534	434	85	1053	1.46	(1.13-1.88)	0.007
**Combined**	**0.31**	**388**	**348**	**82**	**818**	**0.29**	**5428**	**4394**	**932**	**10754**	**1.12**	**(1.00-1.26)**	**0.05**
												(P_{het} = 0.40, I^2= 1.5%)	

**Non-hyperdiploid/ *ETV6/RUNX1***	Case genotypes					Control genotypes							
**rs35837782 (10q26.13, *LHPP*)**	**RAF[a](G)**	**AA**	**AG**	**GG**	**Total**	**RAF(G)**	**AA**	**AG**	**GG**	**Total**	**OR[b]**	**95% CI[c]**	***P*-value**
UK GWAS	0.68	46	171	192	409	0.62	745	2510	1945	5200	1.32	(1.14-1.54)	$2.43x10^{-4}$
German GWAS	0.67	69	253	277	599	0.63	268	952	804	2024	1.04	(0.90-1.20)	0.009
German replication	0.65	113	340	348	801	0.63	204	701	574	1479	1.1	(0.97-1.24)	0.152
UK replication 1	0.63	35	87	93	215	0.62	150	475	405	1030	1.05	(0.85-1.29)	0.671
UK replication 2	0.7	14	115	112	241	0.61	167	500	395	1062	1.54	(1.24-1.91)	8.85E-05
**Combined**	**0.66**	**276**	**966**	**1022**	**2265**	**0.62**	**1534**	**5139**	**4123**	**10795**	**1.16**	**(1.08-1.25)**	**$2.91X10^{-5}$**
												(P_{het} = 0.01, I2= 70%)	
**rs4762284 (12q23.1,*ELK3*)**	**RAF(T)**	**AA**	**AT**	**TT**	**Total**	**RAF(T)**	**AA**	**AT**	**TT**	**Total**	**OR**	**95% CI**	***P*-value**
UK GWAS	0.32	192	171	46	409	0.3	2578	2160	462	5200	1.12	(0.96-1.31)	0.125
German GWAS	0.34	260	271	68	599	0.28	1072	773	179	2024	1.31	(1.15-1.50)	$1.46X10^{-4}$
German replication	0.32	353	366	74	793	0.28	746	619	109	1474	1.22	(1.06-1.40)	0.004
UK replication 1	0.31	102	91	21	214	0.3	497	408	98	1003	1.05	(10.84-1.31)	0.697
UK replication 2	0.35	98	116	25	239	0.29	534	434	85	1053	1.32	(1.07-1.63)	0.009
**Combined**	**0.33**	**1005**	**1015**	**234**	**2254**	**0.29**	**5428**	**4394**	**932**	**10754**	**1.21**	**(1.13-1.30)**	**$1.16X10^{-7}$**
												(P_{het} = 0.33, I^2= 14%)	

Table S4: Relationship between SNP genotype and ALL subtype. RAF: Risk Allele Frequency, OR: Odds Ratio, *P*-value obtained from logistic regression.

		P-values conditional on:		
**SNP**	**Discovery *P*-values***	**rs3731217**	**rs3731249**	**rs77728904**
**rs3731217**	$1.52x10^{-08}$	-	$6.24x10^{-08}$	4.18E-08
**rs3731249***	$6.03x10^{-16}$	$1.54x10^{-12}$	-	3.82E-09
**rs77728904**	$6.26x10^{-07}$	$3.74x10^{-05}$	0.10	-

Table S5: Conditional analysis of SNPs significant in 9p21.3 region and newly claimed independent SNP rs77728904. *P*-values obtained from a meta-analysis of B cell ALL UK and German GWAS series after imputation with UK10k and 1KG reference panels. rs3731249 is the recently described low frequency variant by Vijayakrishnan *et al*. *P*-values* obtained through meta-analysis under a fixed model.

	Study	Controls RAF[a]	Hyperdiploid* (n,RAF)	t(12:21)* (n,RAF)	t(1;19)* (n,RAF)	t(9;22)* (n,RAF)	t(4;11) (n,RAF)	*CDKN2A* deletion[$] (n,RAF)
**rs35837782**	UKGWAS	0.62	0.97 (289, 0.67)	0.29 (126, 0.64)	0.54 (5, 0.70)	0.87 (7, 0.79)	0.40 (2, 0.75)	0
	German GWAS	0.63	0.91 (176, 0.67)	0.35 (63,0.63)	0.53 (5, 0.90)	0.28 (20, 0.70)	0.66 (4, 0.75)	0
	German replication	0.63	0.94 (230, 0.65)	0.24 (339, 0.67)	0.17 (31, 0.56)	0.34 (39, 0.67)	0.26 (12, 0.54)	0
	UK Replication 1	0.62	0.08 (179, 0.70)	0.84 (141, 0.67)	0.50 (15, 0.60)	0.96 (9, 0.67)	0	0.73 (58, 0.66)
	UK Replication 2	0.61	0.95 (182, 0.67)	0.03 (151, 0.62)	0.62 (22, 0.70)	0.66 (14, 0.68)	0	
	Combined *P*-value		0.49	0.46	0.32	0.33	0.16	
**rs4762284**	UKGWAS	0.30	0.22 (289,0.35)	0.74 (126,0.32)	0.50 (5, 0.30)	0.20 (6, 0.50)	0.37 (2, 0.75)	0
	German GWAS	0.28	0.58 (176, 0.32)	0.46 (63, 0.30)	0.99 (5, 0.20)	0.32 (21,0.40)	0.27 (4, 0.38)	0
	German replication	0.28	0.54 (227, 0.32)	0.16 (336, 0.29)	0.97 (31, 0.31)	0.21 (38, 0.27)	0.11 (12, 0.42)	0
	UK Replication 1	0.30	0.79 (180, 0.30)	0.92 (143, 0.31)	0.67 (16, 0.34)	0.09 (9, 0.50)	0	0.94 (52, 0.31)
	UK Replication 2	0.29	0.22 (182, 0.32)	0.40 (150, 0.36)	0.66 (20, 0.38)	0.15 (14, 0.46)	0	
	Combined *P*-value		0.93	0.39	0.55	0.20	**0.03**	

Table S6: Relationship between genotype and specific tumour karyotype. *P*-values are given for case only analysis, along with corresponding numbers (n) and risk allele frequencies (RAF) in parenthesis.*status for selected chromosome anomalies.[$]*CDKN2A* deletion status available for combined UK replication 1 and 2 data sets.

	Hazard ratio	95% confidence interval		*P*
rs35837782 genotype AA[a]	0.89	0.49	1.64	0.711
rs35837782 genotype GG[a]	1.08	0.74	1.57	0.689
rs4762284 genotype AA[a]	0.88	0.60	1.29	0.509
rs4762284 genotype TT[a]	1.24	0.72	2.14	0.437

Table S7. Hazard ratios for an event for rs35837782 and rs4762284 by genotype. [a] in comparison to heterozygous patients.

	Hazard ratio	95% confidence interval		*P*
rs35837782 genotype AA[a]	0.68	0.33	1.42	0.301
rs35837782 genotype GG[a]	1.10	0.73	1.68	0.640
rs4762284 genotype AA[a]	0.88	0.57	1.35	0.555
rs4762284 genotype TT[a]	1.09	0.59	2.04	0.778
Age at diagnosis ≥10 years[b]	1.48	0.95	2.31	0.082
WBC ≥50,000 and <100,000/µl[c]	2.61	1.56	4.38	<0.001
WBC ≥100,000/µl[c]	2.08	1.15	3.75	0.015
ETV6-RUNX1-positivity[d]	0.50	0.16	1.62	0.248
DNA-PCR MRD standard-risk[e]	1.92	1.56	4.38	0.012
DNA-PCR MRD high-risk[f]	4.21	2.55	6.94	<0.001

[a] in comparison to heterozygous patients; [b] in comparison to patients <10 years of age at initial diagnosis; [c] WBC = white blood cell count at diagnosis, in comparison to WBC<50,000/µl at diagnosis; [d] in comparison to *ETV6-RUNX1*-negatives; [e] in comparison to DNA-PCR MRD intermediate- and high-risk; [f] in comparison to DNA-PCR MRD standard- and intermediate-risk

Table S8. Multivariate hazard ratios for an event for rs35837782 and rs4762284 by genotype.

CHR	Pos (hg38)	LD (r^2)	LD (D')	SNP	Promoter histone marks	Enhancer histone marks	DNAse	Proteins bound	Motifs changed	GENCODE genes	dbSNP func annot	CADD scores	GERP scores	RegulomeDB Score
12	96170950	0.24	-0.58	rs2024429		8 tissues	HRT,MUS		7 altered motifs	23kb 5' of ELK3				
12	96190403	0.5	-0.85	rs1558988		BLD	BLD,BLD,MUS		BCL	4kb 5' of ELK3				
12	96192843	0.71	0.88	rs7132101	SKIN	6 tissues	4 tissues		AP-2,HMG-IY	1.5kb 5' of ELK3				
12	96193146	0.46	-0.91	rs12297663	ESDR, SKIN	5 tissues	ESDR,BLD		E2F,ERalpha-a	1.2kb 5' of ELK3				
12	96197874	0.5	-0.89	rs7315748	4 tissues	17 tissues	8 tissues	POL2	6 altered motifs	ELK3	intronic			
12	96198310	0.8	0.96	rs7955113	10 tissues	17 tissues	19 tissues	GATA2, JUND, POL2B		ELK3	intronic			
12	96203803	0.82	0.94	rs2268513		4 tissues			YY1	ELK3	intronic			
12	96204171	0.82	0.94	rs2268511		BLD, SKIN, VAS	BRST,SKIN		6 altered motifs	ELK3	intronic			
12	96206002	0.41	-0.9	rs2268509	BLD	7 tissues	5 tissues			ELK3	intronic			
12	96206378	0.41	-0.9	rs4762283	BLD	5 tissues			ATF3,E2F	ELK3	intronic			
12	96208442	0.83	0.99	rs2887095	BLD	16 tissues	10 tissues		4 altered motifs	ELK3	intronic			
12	96208965	0.83	0.94	rs2268508	FAT, BLD, MUS	20 tissues	15 tissues		6 altered motifs	ELK3	intronic			
12	96211206	0.85	0.99	rs2887096	BLD	15 tissues	VAS,SKIN		4 altered motifs	ELK3	intronic			
12	96213111	0.86	0.99	rs2075362	BLD	16 tissues	5 tissues		SRF	ELK3	intronic			
12	96215566	0.24	-1	rs17736737	10 tissues	17 tissues	22 tissues	7 bound proteins	Crx,Obox3,Pitx2	ELK3	intronic			
12	96217870	0.93	0.99	rs12299943		8 tissues			6 altered motifs	ELK3	intronic			
12	96218316	0.95	1	rs12828252	LNG	11 tissues	SKIN,LNG		PLZF	ELK3	intronic			
**12**	**96218984**	**1**	**1**	**rs4762284**	**SKIN**	**9 tissues**	**LNG,BRST,BRN**		**HMG-IY,Mef2,TATA**	**ELK3**	**intronic**	**4.501**	**1.88**	**3a**
12	96220347	1	1	rs2268507	7 tissues	14 tissues	8 tissues		7 altered motifs	ELK3	intronic			
12	96220447	1	1	rs2268506	LNG	15 tissues			5 altered motifs	ELK3	intronic			
12	96221038	0.6	-1	rs11837546	MUS	18 tissues	17 tissues			ELK3	intronic			
12	96222273	0.85	0.94	rs1030137						ELK3	intronic			
12	96223246	0.51	-0.84	rs2300554		12 tissues	MUS,MUS,SKIN		AP-1,Pax-4	RP11-394J1.2	intronic			
12	96223526	0.69	0.97	rs2302902		13 tissues	GI			RP11-394J1.2	intronic			
12	96224030	0.69	0.97	rs4762138		11 tissues	10 tissues	BATF, NFKB	Pou2f2	ELK3	intronic			
12	96224128	0.69	0.98	rs4762139		11 tissues	11 tissues	BATF	8 altered motifs	ELK3	intronic			
12	96224985	0.23	-0.91	rs2300553	BRN	13 tissues	5 tissues		5 altered motifs	ELK3	intronic			
12	96228656	0.3	0.61	rs10777775			SKIN		4 altered motifs	ELK3	intronic			
12	96229248	0.61	0.93	rs11108446		8 tissues	11 tissues		Hoxb8,Hsf,Sox	ELK3	intronic			
12	96230342	0.6	0.92	rs9971896		FAT, BRST, SKIN			4 altered motifs	ELK3	intronic			
12	96232578	0.27	0.69	rs9971785		4 tissues			18 altered motifs	ELK3	intronic			
12	96236848	0.28	0.64	rs10777776			BLD,BLD,BRN	CTCF		ELK3	intronic			

12	96236970	0.21	0.84	rs7975154	4 tissues	16 tissues	9 tissues		5 altered motifs	ELK3	intronic			
12	96237097	0.2	0.84	rs7978130	5 tissues	16 tissues	15 tissues		Ets	ELK3	intronic			
12	96258192	0.23	0.77	rs2268502		22 tissues	21 tissues		Maf,NF-E2,SIX5	ELK3	intronic			
12	96258580	0.26	0.54	rs10745751			20 tissues		Foxa	ELK3	intronic			
12	96260907	0.27	0.55	rs7138031		6 tissues	5 tissues	POL2,<br>POL2B		ELK3	intronic			
12	96264612	0.3	0.59	rs2300550		6 tissues	BLD,VAS,SKIN	POL2B		ELK3	intronic			
12	96266933	0.29	0.59	rs7974921		SKIN	BLD,VAS	POL2B	HNF1,Hoxa4,Prrx2	ELK3	intronic			
12	96277913	0.29	0.58	rs10777780					Fox,Foxi1,Pou5f1	347bp 3' of CDK17				
12	96283165	0.24	0.51	rs61938858		GI, HRT			Hsf,SRF	CDK17	intronic			
12	96290592	0.28	0.58	rs7485280					Pou5f1,RFX5	CDK17	intronic			
12	96291444	0.29	0.58	rs10860011					Gm397,Mtf1,Pax-5	CDK17	intronic			
12	96293047	0.28	0.58	rs11108455					6 altered motifs	CDK17	intronic			
12	96298065	0.25	0.53	rs4762295					HES1,RXRA	CDK17	intronic			
12	96300924	0.25	0.53	rs139772832		BRN, LNG			9 altered motifs	CDK17	intronic			
12	96301550	0.32	0.67	rs10860013						CDK17	intronic			
12	96301660	0.29	0.58	rs10777782					5 altered motifs	CDK17	intronic			
12	96302216	0.29	0.58	rs9971815		BRN, VAS			DMRT2,GATA,Sox	CDK17	intronic			
12	96303823	0.28	0.57	rs12313403		7 tissues	10 tissues	RAD21	GR	CDK17	intronic			
12	96304859	0.25	0.53	rs10777783					Cdx	CDK17	intronic			
12	96314497	0.26	0.58	rs10745753			ADRL,HRT,BLD		ERalpha-a,RORalpha1	CDK17	intronic			
12	96322285	0.25	0.56	rs10777784					Mef2	CDK17	intronic			
12	96334248	0.26	0.57	rs9308300		6 tissues			Mef2,Zfp105	CDK17	intronic			
12	96343062	0.22	0.48	rs11108475		BLD, VAS	BLD		7 altered motifs	CDK17	intronic			
12	96344775	0.22	0.48	rs10860019				KAP1	Pax-4	CDK17	intronic			
12	96347530	0.25	0.51	rs11503397	5 tissues	11 tissues	10 tissues		ATF3,AhR	CDK17	intronic			
12	96347598	0.24	0.49	rs61939062	5 tissues	11 tissues	5 tissues		14 altered motifs	CDK17	intronic			
12	96348605	0.24	0.54	rs10745758			SKIN			CDK17	intronic			
12	96353932	0.22	0.52	rs10860021			BLD,LNG		AP-1,Pax-8,TBX5	CDK17	intronic			
12	96357513	0.22	0.52	rs7973336		10 tissues	GI,SKIN		CDP,HNF1	CDK17	intronic			
12	96361319	0.24	0.54	rs7974022	BLD, GI	15 tissues	10 tissues		5 altered motifs	CDK17	intronic			
12	96365296	0.23	0.53	rs10860024						CDK17	intronic			
12	96369430	0.24	0.53	rs10860025					15 altered motifs	CDK17	intronic			
12	96373379	0.24	0.53	rs3925974		15 tissues			7 altered motifs	CDK17	intronic			
12	96377611	0.23	0.51	rs10860028			9 tissues		5 altered motifs	CDK17	intronic			
12	96378257	0.23	0.51	rs1583793	BLD	BLD, GI, VAS	5 tissues		BDP1	CDK17	intronic			

12	96385148	0.22	0.51	rs10860030					Mef2	CDK17	intronic			
12	96390447	0.24	0.52	rs7305520		4 tissues			Eomes,TBX5,ZEB1	CDK17	intronic			
12	96392259	0.24	0.53	rs10437881			VAS		Foxj2	CDK17	intronic			
12	96394581	0.24	0.54	rs6538709		BLD, VAS	BLD,MUS		HNF4,NRSF	CDK17	intronic			
12	96398788	0.22	0.54	rs7968046	23 tissues	ESDR, IPSC, ESC	32 tissues	POL2B		CDK17	intronic			
10	124560664	0.36	-0.66	rs7915230	4 tissues	16 tissues	16 tissues	GR	MOVO-B,VDR	LHPP	intronic			
10	124564186	0.27	-0.87	rs11245292		HRT			7 altered motifs	LHPP	intronic			
10	124565056	0.27	-0.87	rs10901760						LHPP	intronic			
10	124565875	0.27	-0.86	rs7090456	ESC, MUS	17 tissues	21 tissues	4 bound proteins	16 altered motifs	LHPP	intronic			
10	124570770	0.27	-0.87	rs3891517		BLD			Mrg1::Hoxa9	LHPP	intronic			
10	124570862	0.27	-0.87	rs956692		BLD	BLD		7 altered motifs	LHPP	intronic			
10	124571199	0.28	-0.89	rs10794153					CCNT2,GATA	LHPP	intronic			
10	124571717	0.27	-0.87	rs12269523		BLD				LHPP	intronic			
10	124571942	0.27	-0.87	rs72837376		BLD	BLD		CTCF,RREB-1,VDR	LHPP	intronic			
10	124572495	0.27	-0.87	rs12358685					8 altered motifs	LHPP	intronic			
10	124573497	0.27	-0.87	rs10794155					Mef2,PLZF,ZEB1	LHPP	intronic			
10	124573884	0.27	-0.86	rs10901766					4 altered motifs	LHPP	intronic			
10	124574339	0.28	-0.86	rs4962382		SKIN, BRN			8 altered motifs	LHPP	intronic			
10	124574516	0.28	-0.88	rs4962383		IPSC, THYM			BHLHE40,NF-E2,NRSF	LHPP	intronic			
10	124574585	0.29	-0.89	rs4962384		IPSC, THYM			4 altered motifs	LHPP	intronic			
10	124574675	0.29	-0.89	rs56260700		IPSC			GATA,Gfi1	LHPP	intronic			
10	124574696	0.28	-0.88	rs55762237		IPSC			8 altered motifs	LHPP	intronic			
10	124575333	0.29	-0.88	rs10901767					AP-4,E2A,LBP-1	LHPP	intronic			
10	124575463	0.29	-0.88	rs10901768					GR	LHPP	intronic			
10	124576807	0.23	-0.89	rs4962658					7 altered motifs	LHPP	intronic			
10	124576960	0.37	-0.88	rs10901770					SP1	LHPP	intronic			
10	124577264	0.31	-0.83	rs10751596					BRCA1,GATA	LHPP	intronic			
10	124577503	0.37	-0.88	rs12250595		BLD, THYM	BRN		5 altered motifs	LHPP	intronic			
10	124579799	0.27	-0.89	rs10901771			HRT,MUS			LHPP	intronic			
10	124579802	0.29	-0.79	rs10901772			HRT,MUS		Pax-5	LHPP	intronic			
10	124590335	0.33	-0.88	rs10901776			6 tissues	CTCF	BDP1,Pax-6,Spz1	LHPP	intronic			
10	124592024	0.39	-0.95	rs4962664					Maf,Nkx2	LHPP	intronic			
10	124592104	0.34	-0.88	rs4962665		BLD			GATA,KAP1,p53	LHPP	intronic			
10	124594058	0.34	-0.88	rs10901777			ADRL		4 altered motifs	LHPP	intronic			
10	124595400	0.2	-0.96	rs4962666		BLD, THYM				LHPP	intronic			

10	124595640	0.33	-0.76	rs4962667		BLD, THYM	5 tissues	TAL1	4 altered motifs	LHPP	intronic			
10	124595767	0.22	0.97	rs34496154		BLD, THYM	5 tissues		LUN-1,SRF	LHPP	intronic			
10	124595846	0.22	0.97	rs61870251		BLD, THYM	4 tissues		Nanog	LHPP	intronic			
10	124595931	0.2	-0.96	rs4962668		BLD, THYM	BLD		Maf	LHPP	intronic			
10	124596868	0.21	-0.98	rs11245308	LIV	5 tissues	IPSC,BLD		Nkx3,OTX	LHPP	intronic			
10	124597368	0.36	-0.89	rs1123987		7 tissues	HRT,GI		6 altered motifs	LHPP	intronic			
10	124598237	0.22	0.99	rs34042290		4 tissues	THYM		NF-kappaB	LHPP	intronic			
10	124598536	0.34	-0.85	rs731622		4 tissues	VAS		6 altered motifs	LHPP	intronic			
10	124599410	0.38	-0.94	rs10901778			BLD		TATA	LHPP	intronic			
10	124599835	0.38	-0.94	rs1563282		5 tissues	BLD,BLD,BLD			LHPP	intronic			
10	124600025	0.38	-0.94	rs1123988		5 tissues	THYM		GR,Pax-5,STAT	LHPP	intronic			
10	124600888	0.21	-0.98	rs11599414		7 tissues	BLD,ADRL,THYM		BCL,CDP,NF-kappaB	LHPP	intronic			
10	124601269	0.39	-0.95	rs10901779			BLD,BLD,BLD		7 altered motifs	LHPP	intronic			
10	124602481	0.21	-0.98	rs9783260		6 tissues			7 altered motifs	LHPP	intronic			
10	124602776	0.21	-0.98	rs9783263		7 tissues	BLD		10 altered motifs	LHPP	intronic			
10	124603049	0.4	-0.96	rs10901780			BLD		HDAC2,PLAG1,TLX1::NFIC	LHPP	intronic			
10	124603787	0.28	0.94	rs2885520	LIV	8 tissues	ESC		AP-2,NF-I,NRSF	LHPP	intronic			
10	124603928	0.34	0.79	rs2362511	LIV	9 tissues	5 tissues		12 altered motifs	LHPP	intronic			
10	124604086	0.84	0.95	rs12779301	LIV	9 tissues	THYM,BLD		CTCF,RBP-Jkappa	LHPP	intronic			
10	124604169	0.84	0.95	rs12779515	LIV	9 tissues	MUS			LHPP	intronic			
10	124604199	0.21	-0.98	rs9783250	LIV	9 tissues			5 altered motifs	LHPP	intronic			
10	124604391	0.38	-0.96	rs10901781			BLD,SKIN,THYM	GATA2	NRSF,Rad21	LHPP	intronic			
**10**	**124604740**	**1**	**1**	**rs35837782**	**BRN**	**10 tissues**	**7 tissues**		**Hic1**	**LHPP**	**intronic**	**1.284**	**-4.5**	**5**
10	124604790	0.89	0.99	rs12765129	BRN	10 tissues	7 tissues		4 altered motifs	LHPP	intronic			
10	124605150	0.38	0.85	rs11812870	BRN	11 tissues	7 tissues		4 altered motifs	LHPP	intronic			
10	124605409	0.42	-1	rs3740541	ESDR, BRN, LIV	9 tissues	BLD,THYM		NF-kappaB,Rad21	LHPP	intronic			
10	124605598	0.22	0.99	rs3740540	BRN, LIV	10 tissues	ESDR		ERalpha-a,Mrg	LHPP	intronic			
10	124605715	0.74	0.88	rs12771808	ESDR, BRN, LIV	8 tissues	KID			LHPP	intronic			
10	124605790	0.52	0.99	rs10901782			4 tissues		BCL,Ik-2,STAT	LHPP	intronic			
10	124605911	0.88	0.99	rs12777136	BRN, LIV	9 tissues	6 tissues	CMYC,MAX	ATF3,ERalpha-a,HNF4	LHPP	intronic			
10	124605932	0.42	-0.99	rs10901783			7 tissues	CMYC,MAX	5 altered motifs	LHPP	intronic			
10	124606098	0.52	0.99	rs4962391	BLD, BRN, LIV	10 tissues	5 tissues		KAP1	LHPP	intronic			
10	124606304	0.2	-0.96	rs4962392	BLD, BRN, LIV	11 tissues	4 tissues	4 bound proteins	EWSR1-FLI1,NRSF,Pbx3	LHPP	intronic			
10	124606497	0.49	0.95	rs11307680	BRN, LIV	12 tissues	4 tissues	HNF4A,<br>HNF4G,<br>POL2	17 altered motifs	LHPP	intronic			

10	124606694	0.41	-0.99	rs11245312	BRN, LIV	12 tissues	BLD,THYM		GR,NRSF	LHPP	intronic			
10	124606842	0.51	0.97	rs7907954	BRN, LIV	14 tissues	KID,GI,LIV		5 altered motifs	LHPP	intronic			
10	124606936	0.21	-0.98	rs11594077	BRN, LIV	14 tissues	5 tissues		Pax-5	LHPP	intronic			
10	124606939	0.21	0.97	rs35986134	BRN, LIV	14 tissues	5 tissues			LHPP	intronic			
10	124606986	0.88	0.99	rs7923479	BRN, LIV	14 tissues	5 tissues		8 altered motifs	LHPP	intronic			
10	124607077	0.74	-0.99	rs10901785			7 tissues		6 altered motifs	LHPP	intronic			
10	124607212	0.41	-0.98	rs11245313	BRN, LIV	11 tissues	BLD,THYM,LIV		5 altered motifs	LHPP	intronic			
10	124607521	0.21	0.97	rs12761008	BRN	6 tissues	BRN			LHPP	intronic			
10	124607814	0.21	-0.98	rs2061069	BRN	4 tissues	IPSC		NRSF,PU.1	LHPP	intronic			
10	124607842	0.21	-1	rs2061070	SKIN, BRN	5 tissues	IPSC		4 altered motifs	LHPP	intronic			
10	124608056	0.28	-0.76	rs12249375	BRN	9 tissues	5 tissues		9 altered motifs	LHPP	intronic			
10	124608639	0.56	0.89	rs11245314		7 tissues		USF1	4 altered motifs	LHPP	intronic			
10	124608691	0.24	-0.75	rs67233244		7 tissues			18 altered motifs	LHPP	intronic			
10	124609101	0.22	0.96	rs11245315		4 tissues			DMRT1,DMRT7	LHPP	intronic			
10	124609616	0.21	-0.66	rs11245316		BLD, BRN, THYM			Klf7	LHPP	intronic			
10	124609777	0.21	-0.66	rs10901787			OVRY	CTCF	Irf	LHPP	intronic			
10	124611031	0.35	-0.66	rs12355969		4 tissues	THYM		HDAC2,NRSF,Pou2f2	LHPP	intronic			
10	124611427	0.23	-0.68	rs66631105	BLD	11 tissues	5 tissues		Sin3Ak-20,WT1	LHPP	intronic			

Table S9: Epigenetic annotation of genome-wide significant SNPs. Data are shown for query SNPs and their proxies ($r^2 > 0.2$ in 1000 Genomes EUR Phase 1 data) demonstrating evidence of histone marks, DNase hypersensitivity sites or transcription factor occupancy in RegulomeDB analysis. Also indicated are genomic evolutionary rate profiling (GERP) scores and combined annotation dependent depletion (CADD) scores. Chr, chromosome; DNAse HS, DNAse hypersensitivity; Fre, frequency; GERP, Genomic Evolutionary Rate Profiling; LD, linkage disequilibrium; Pos, position; CADD, combined annotation dependent depletion. RegulomeDB scores: 2b, TF binding + any motif + Dnase Footprint + Dnase peak; 4, TF binding + DNAse peak; 5, TF binding or Dnase peak; 6, other binding or Dnase peak.

P-value	SNP	SNP Chr.	SNP Chr. Position	Probe	Probe Chr.	Probe Chr. position	SNP Alleles	Minor Allele	Z-score	Gene name	*FDR
6.85E-04	rs4762284	12	95136893	3420014	12	95181371	T/A	T	-3.4	ELK3	0.2

Table S10: eQTL analysis in LCL and blood tissues for rs4762284 using the eQTL blood browser online resources. Genes within 1 Mb of query SNP were assessed for association. *FDR: False discovery rate values shown are from 'probe level' rates as described by Westra H. *et al*, Nat.Gen (2012).

SNP Oligo name/Allele	Sequence
rs12751998_ALG	GAAGGTGACCAAGTTCATGCTATTTACAGTTGTCTACTCCCCG
rs12751998_ALT	GAAGGTCGGAGTCAACGGATTCATTTACAGTTGTCTACTCCCCCT
rs12751998_C1	ACTGGGAGCAAAAGAAGATGAGAGAAAA
rs890579_ALC	GAAGGTGACCAAGTTCATGCTGGTATTTTAAAAATAATCCATGAAAAACAATGG
rs890579_ALT	GAAGGTCGGAGTCAACGGATTGGTATTTTAAAAATAATCCATGAAAAACAATGA
rs890579_C1	CCCAGGGTGCAGATTTACTCTCTTT
rs74952692_ALC	GAAGGTGACCAAGTTCATGCTGTGCCTTGCATCCCCTTAGC
rs74952692_ALT	GAAGGTCGGAGTCAACGGATTGTGCCTTGCATCCCCTTAGT
rs74952692_C2	GCAGCCTCAGGAAACTTACAGTCAT
rs16852911_ALA	GAAGGTGACCAAGTTCATGCTAGCAGAAATGCCACGAATACAGAGA
rs16852911_ALT	GAAGGTCGGAGTCAACGGATTAGCAGAAATGCCACGAATACAGAGT
rs16852911_C1	TTCCTACTGTTTCTGGGAACTTAGACTTT
rs145666495_ALG	GAAGGTGACCAAGTTCATGCTACCGCCCCAGTAAAGCCCG
rs145666495_ALT	GAAGGTCGGAGTCAACGGATTCTACCGCCCCAGTAAAGCCCT
rs145666495_C1	TCAGTGCCTTAAGAAAGGCAGCCT
rs6933843_ALG	GAAGGTGACCAAGTTCATGCTAAGGAGAAAAGAGAAAGTATGAAAAAGTAG
rs6933843_ALT	GAAGGTCGGAGTCAACGGATTCAAGGAGAAAAGAGAAAGTATGAAAAAGTAT
rs6933843_C1	AGATCCCTAATGCTGTCTGGTCCAT
rs62579834_ALA	GAAGGTGACCAAGTTCATGCTGCATGAACAGCCTGCTCAGAACA
rs62579834_ALC	GAAGGTCGGAGTCAACGGATTCATGAACAGCCTGCTCAGAACC
rs62579834_C1	TGCAGTTGAAGCATGAGCTGAGGAT
rs35837782_ALA	GAAGGTGACCAAGTTCATGCTCCCTCTGATGCCAGTGCCCA
rs35837782_ALG	GAAGGTCGGAGTCAACGGATTCCTCTGATGCCAGTGCCCG
rs35837782_C1	GGGGCGGCTGCCCAGTGAA
rs16937230_ALC	GAAGGTGACCAAGTTCATGCTGGTGCTCACGTCCCTGG
rs16937230_ALT	GAAGGTCGGAGTCAACGGATTGCTGGTGCTCACGTCCCTGA
rs16937230_C1	GTCTCCACAGAGGAGGAGGACAT
rs1001205_ALC	GAAGGTGACCAAGTTCATGCTACTGCTCTGCAGATAGGCTAGG
rs1001205_ALT	GAAGGTCGGAGTCAACGGATTCACTGCTCTGCAGATAGGCTAGA
rs1001205_C1	CAGCCTTGGCTGAACCAACCCTT
rs1419981_ALA	GAAGGTGACCAAGTTCATGCTCATCAGACATCAGCCCAGGGT
rs1419981_ALG	GAAGGTCGGAGTCAACGGATTCATCAGACATCAGCCCAGGGC
rs1419981_C1	GTCAGGCAATGGCAATTACTCAGCT
rs4762284_ALA	GAAGGTGACCAAGTTCATGCTCACATAAAAGGCATGCTAGAAATCTTTTTTTT
rs4762284_ALT	GAAGGTCGGAGTCAACGGATTCACATAAAAGGCATGCTAGAAATCTTTTTTTA
rs4762284_C1	GCGCCCGGCCAAGATAAGCATT
rs7152152_ALC	GAAGGTGACCAAGTTCATGCTTTGGGGGAACAGGGACAATG
rs7152152_ALT	GAAGGTCGGAGTCAACGGATTCCTTTGGGGGAACAGGGACAATA
rs7152152_C2	GCACCCTGGTTGGTACATGGTTAAT
rs10148274_ALA	GAAGGTGACCAAGTTCATGCTAATTTCAAAAACTGGAAAATTTACGAATGAAAT
rs10148274_ALC	GAAGGTCGGAGTCAACGGATTAATTTCAAAAACTGGAAAATTTACGAATGAAAG
rs10148274_C2	GAAGCCCGATTTTGGAAATCAGATCTTAA
rs4448919_ALA	GAAGGTGACCAAGTTCATGCTTCCACACTGAGCCTTAAGTTGGTA
rs4448919_ALG	GAAGGTCGGAGTCAACGGATTCCACACTGAGCCTTAAGTTGGTG
rs4448919_C1	CCATCAATATATTGCAGCGAGACAATGAA
rs10411772_ALA	GAAGGTGACCAAGTTCATGCTCCTCTGCCTTCACCTTCCAACA
rs10411772_ALC	GAAGGTCGGAGTCAACGGATTCTCTGCCTTCACCTTCCAACC
rs10411772_C1	TTAATGACAGGGTCCTGGCTCTCAT
rs12751998_F	TCCTGTTTGTAACACCACTTTCC

rs12751998	TGCCTGGCTATTTTATCCCTCG
rs890579_F	CAGCACTGGTAGAGGCTGAC
rs890579	GGATGACAGAGAACAAGATTAGACA
rs74952692_F	TTTCCTATGCTGTCCACCCT
rs74952692	ACAACCCTTACCACACCTCA
rs16852911_F	GGAGACCCTCTCCACAGTGA
rs16852911_R	GCACAGCAAGTGGGAAAGTG
rs145666495_F	GCCCAACAATGCCCTCAAAT
rs145666495_R	CAAGTGGCAAGCTCAGGAAG
rs3748079_F	GGGTCAGCAGCTGTCTCTTT
rs3748079_R	CAGAGCACCCAAGCGCTAA
rs6933843_F	TGCTGGGACATGTGGAAGTC
rs6933843_R	AGCATTTAGGGAGACAATGGGA
rs62579834_F	TGTGGCCATAACTACTCAGCA
rs62579834_R	TTCCCCAGACCTCGTTGTAC
rs35837782_F	CACGTGGACAGCAGGGAAC
rs35837782_R	GGGGCAGAGACTCTCACAAC
rs16937230_F	CCGCGTCACCCATCTAATGA
rs16937230_R	GGGCCTGGATTCTCCCTCTT
rs1001205_F	TCGTGTTCCTGGAGGAGAGAA
rs1001205_R	TTCCTCCTGGATCTGGCTCA
rs1419981_F	TTCTGTTTCCCCTAGGCTGG
rs1419981_R	AGCACAAGTCCTGGTCCAAT
rs4762284_F	TTGGGTGGTGGGTATGCTAG
rs4762284_R	CATGGAGGCCTGCTCTAAGT
rs7152152_F	CTTGCTTCCAGTGGTGTCCC
rs7152152_R	GCCCTTCAAGACAGTCCAGG
rs10148274_F	TGATCCCCTCTCCTATGCAC
rs10148274_R	GCTGTCTTGTAATGCACCCG
rs4448919_F	TCGGGACCCTGACTGTTTC
rs4448919_R	TGTGGTTCTGGCAGAGTAGG
rs10411772_F	CGCCAGTCACATCCACTCAT
rs10411772_R	TCCTGAGGCTTCCCGTTACT

Table S11: Oligonucleotides used in this study for KASPAR genotyping and Sanger sequencing.
